# Mechanisms Behind the Pharmacological Application of Biochanin-A: A review

**DOI:** 10.12688/f1000research.126059.3

**Published:** 2023-12-11

**Authors:** P.V. Anuranjana, Fathima Beegum, Divya K.P, Krupa Thankam George, G.L. Viswanatha, Pawan G. Nayak, Abhinav Kanwal, Anoop Kishore, Rekha R. Shenoy, K. Nandakumar

**Affiliations:** 1 Department of Pharmacology, Manipal College of Pharmaceutical Sciences, Manipal Academy of Higher Education, Manipal, Karnataka, 576104, India; 2 Independent Researcher, Bengaluru, Karnataka, India; 3 Department of Pharmacology, All India Institute of Medical Sciences, Bathinda, Punjab, India

**Keywords:** Biochanin-A, Isoflavones, Mechanism, Cancer, Diabetes, Neuroprotection, Cardiovascular, Anti-oxidant

## Abstract

This review was aimed at summarizing the cellular and molecular mechanisms behind the various pharmacological actions of biochanin-A. Many studies have been reported claiming its application in cancers, metabolic disorders, airway hyperresponsiveness, cardiac disorders, neurological disorders, etc. With regard to hormone-dependent cancers like breast, prostate, and other malignancies like pancreatic, colon, lung, osteosarcoma, glioma that has limited treatment options, biochanin-A revealed agreeable results in arresting cancer development. Biochanin-A has also shown therapeutic benefits when administered for neurological disorders, diabetes, hyperlipidaemia, and other chronic diseases/disorders. Isoflavones are considered phenomenal due to their high efficiency in modifying the physiological functions of the human body. Biochanin-A is one among the prominent isoflavones found in soy (glycine max), red clover (Trifolium pratense), and alfalfa sprouts, etc., with proven potency in modulating vital cellular mechanisms in various diseases. It has been popular for ages among menopausal women in controlling symptoms. In view of the multi-targeted functions of biochanin-A, it is essential to summarize it's mechanism of action in various disorders. The safety and efficacy of biochanin-A needs to be established in clinical trials involving human subjects. Biochanin-A might be able to modify various systems of the human body like the cardiovascular system, CNS, respiratory system, etc. It has shown a remarkable effect on hormonal cancers and other cancers. Many types of research on biochanin-A, particularly in breast, lung, colon, prostate, and pancreatic cancers, have shown a positive impact. Through modulating oxidative stress, SIRT-1 expression, PPAR gamma receptors, and other multiple mechanisms biochanin-A produces anti-diabetic action. The diverse molecular mechanistic pathways involved in the pharmacological ability of biochanin-A indicate that it is a very promising molecule and can play a major impact in modifying several physiological functions.

## 1. Introduction

Traditionally, going back to nature has always been an answer, and in reality, natural products have been essential in the development of new drugs, particularly for infectious and cancerous disorders ( Atanasov *et al.*, 2021). Natural products have been a rich source of new drugs for centuries. ‘Anticancer drugs such as Taxol ( *Taxus brevifolia*) and Vinblastine ( *Catharanthus roseus*) and antimalarial drugs such as quinine ( *Cinchona* spp.) and Artemisinin (Artemisia annua)’ were all discovered from natural products and have been effective in treating these diseases ( Singh *et al.*, 2021).

Phytoestrogens are naturally occurring nonsteroidal phenolic plant compounds that can be categorized into flavonoids and non-flavonoids ( Boyle *et al.*, 2003). Flavonoids encompass isoflavones, coumestans, and prenylflavonoids, while non-flavonoids comprise lignans. The major isoflavones are genistein (7,4′-dihydroxy-6-methoxyisoflavone), daidzein (7,4′-dihydroxyisoflavone), glycitein (7,4′-dihydroxy-6-methoxyisoflavone), biochanin-A (5,7-dihydroxy-4′-methoxyisoflavone), and formononetin (7-hydroxy-4′-methoxyisoflavone). Isoflavones, renowned for their estrogenic properties, are predominantly found in legumes from the Fabaceae family ( Dixon and Sumner, 2003), with soybean (Glycine max) as a source of daidzein, genistein, glycitein, and red clover (Trifolium pratense) as a source of formononetin and biochanin-A.

Isoflavones predominantly found in soybean and soybean products serve as major dietary sources for humans. Isoflavones are recognized for their chemoprotective properties, offering an alternative therapeutic approach for various hormonal disorders, including breast cancer, prostate cancer, cardiovascular diseases, osteoporosis, and menopausal symptoms ( Křížová *et al.*, 2019). Biochanin-A, a phytochemical obtained from soy, alfalfa sprouts, red clover plants, chickpeas, etc., has recently gained attention in research due to its various pharmacological applications ( Ferrer and Thurman, 2013). It can benefit humans in various systems. Biochanin-A has been tested for its effect in various cancers, inflammation, osteoarthritis, metabolic disorders, cardiovascular diseases, anti-oxidant properties, hormone-dependent diseases, etc ( Sehdev, Lai and Bhushan, 2009; Kole *et al.*, 2011; Szliszka *et al.*, 2013; Bhardwaj *et al.*, 2014; D. Q. Wu *et al.*, 2014). Biochanin-A has been shown to have a potential neuroprotective impact by modulating multiple critical neurological pathways. Further, biochanin-A is a chief phytoconstituent of the red clover plant, which is well known for alleviating menopause symptoms through its oestrogenic and antioxidant properties ( Romm *et al.*, 2010; Raheja *et al.*, 2018). Patients with prostate and breast malignancies have proven to show a defensive effect from an isoflavone-rich diet with evidence to various epidemiological studies and the mechanism behind such an action is by isoflavone and oestrogen receptor binding resulting in osteoprotective actions ( Messina and Hilakivi-Clarke, 2009; Shu *et al.*, 2009; Lecomte *et al.*, 2017). Common dietary mixtures have gained consideration because of their synergistic impacts with several anticancer drugs seen in different kinds of malignancy. The pleiotropic effects of isoflavones on tumour cells work through modulation of various cellular signalling pathways. As indicated by various in vitro investigations, genistein is more powerful than biochanin-A as far as both oestrogenic activity and cancer prevention ability are concerned ( Ullah *et al.*, 2009). However, biochanin-A is effectively changed over to genistein in first-pass digestion, and genistein can be identified in human plasma after treatment with biochanin-A ( Setchell *et al.*, 2001).

Administration of isoflavones is known to produce a significant pharmacokinetic problem identified with their deprived bioavailability ( Passamonti *et al.*, 2009). Isoflavones are non-nutrient plant components and a subclass of flavonoids. They principally exist as β-glucosides ( Panche, Diwan and Chandra, 2016). Higher content of biochanin-A is found in red clover plants ( Lemežienė *et al.*, 2015). The harmful clastogenic effect presented in genistein is less exhibited in biochanin-A, adding to its more clinical acceptance ( Snyder and Gillies, 2002; Michael McClain *et al.*, 2006). Mutagenicity related to geinstein use is not presented in biochanin-A associated studies. The low incidence of cancer exhibited in the Asian population has been a widely discussed topic globally ( Jin *et al.*, 2016; Tran *et al.*, 2018). It has a close connection to the soy-rich diet of the population ( Hawrylewicz, Zapata and Blair, 1995; He and Chen, 2013; Wei *et al.*, 2017; Ziaei and Halaby, 2017). Soy isoflavones have significance over selective oestrogen receptor modulators (SERMs) and hormone replacement in breast cancer treatment as they possess oestrogen-like rings in their structure ( Sarkar *et al.*, 2009). Though there are no conclusive reports, isoflavones are preferred by women due to their best safety profile and improved quality of life in comparison with hormone replacement therapy to treat menopausal symptoms ( Franco *et al.*, 2016; Ahsan and Mallick, 2017; Chen, Ko and Chen, 2019). Thus, this review was focused on summarizing the pharmacological applications of biochanin-A along with the various cellular and molecular pathways involved in it. The deprived water solubility along with minimal oral bioavailability confined the application of biochanin-A as a drug molecule. Nano-sized biochanin-A phospholipid complex “nBCA-PLCs” have the potential to overcome this limitation and boost its oral bioavailability. When compared to other formulations such as normal biochanin-A phospholipid complex and the suspension of biochanin-A, nBCA-PLCs have relatively higher bioavailability ( Singh *et al.*, 2021). There is compelling evidence that biochanin-A is a bioactive compound with a wide range of biological and pharmacological activities. To help understand the beneficial and myriad therapeutic effects of biochanin-A, our review evaluated past and current findings of the literature and proposed molecular mechanisms behind various disorders such as different types of cancers, diabetes, airway and cardiovascular disorders, neurological disorders etc.

## 2. Biochanin-A and its pharmacological benefits

### 2.1 Cancer

**Figure 1.**
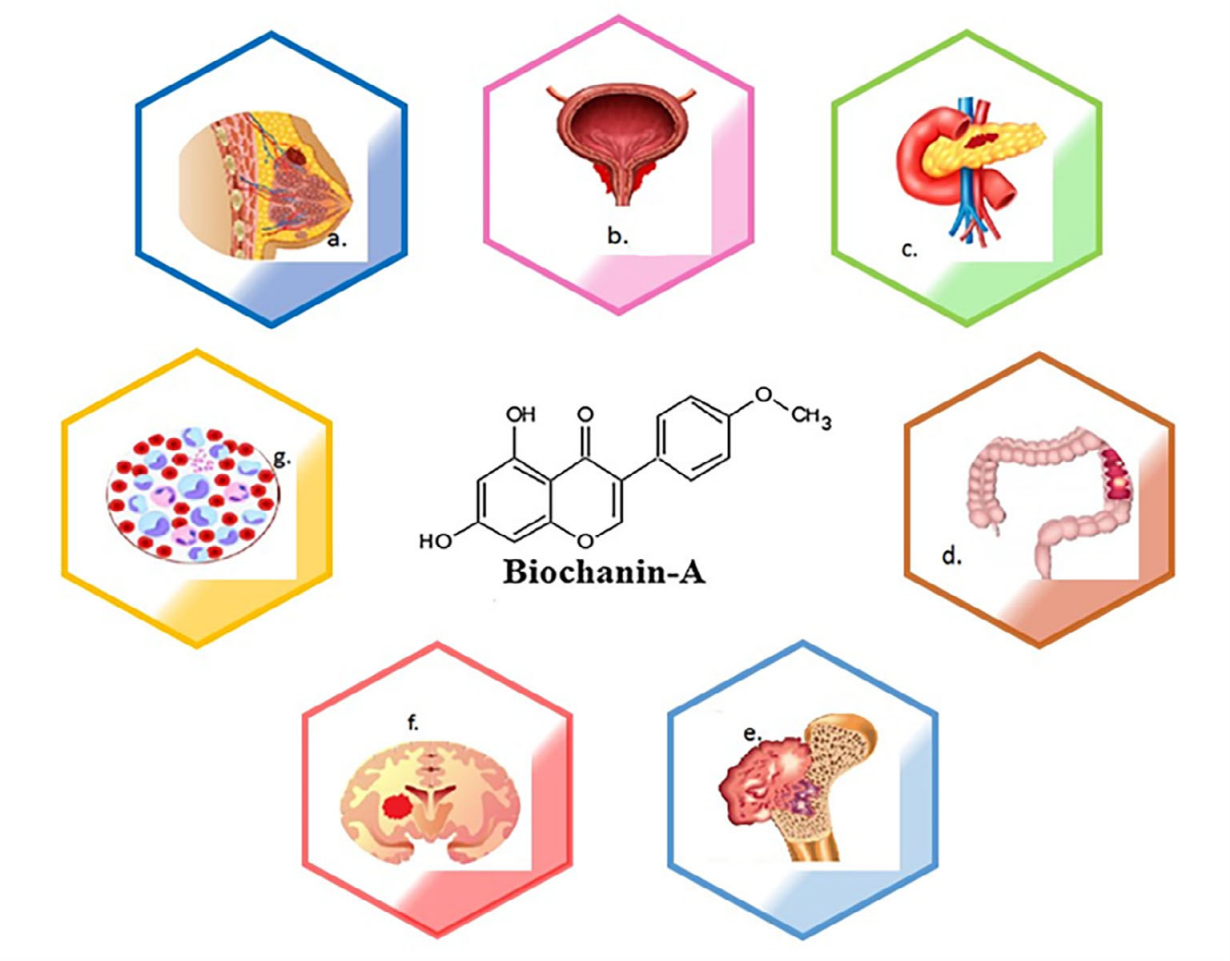
The schematic diagram represents pharmacological actions of biochanin-A in different types of cancer. Biochanin-A has prevented the development of different types of cancers. a) breast cancer, b) prostate cancer, c) lung cancer, d) pancreatic cancer, e) colon cancer, f) osteosarcoma, g) glioma, h) leukaemia. The molecular structure of biochanin-A is depicted in the centre of diagram.

Biochanin-A has prevented/inhibited the development of different types of cancers. Figure 1 depicts the types of cancers where biochanin-A has an impact in controlling the disease. Various kinds of cancer are a) breast cancer, b) prostate cancer, c) lung cancer, d) pancreatic cancer, e) colon cancer, f) osteosarcoma, g) glioma, h) leukaemia. The molecular structure of biochanin-A is depicted in the centre of diagram ( Feng and Lai, 2023).

**2.1.1 Breast cancer**

Breast cancer is the most widely reported form of cancer, presented with several subtypes and varying vulnerability to anti-cancer agents. Tumour cells have shown a unique pattern in terms of uncontrolled growth, dedifferentiated morphology, and resistance to apoptosis ( Baba and Câtoi, 2007). In most types of solid cancers like breast cancer, the normal signalling pathways are interrupted, stimulating refractory growth, no cell death, and progressive invasion of neighbouring tissues. Soy-rich diets in controlling hormone-dependent cancers have gained wide attention lately ( Shu *et al.*, 2009; Kang *et al.*, 2010; Varinska *et al.*, 2015). Good levels of isoflavones in serum serve as protection from the risk of breast cancer ( Kang *et al.*, 2010). Various studies have been tested for the effect of biochanin-A being one of the most beneficial constituents of red clover and soy isoflavones on breast cancer. Unlike chemical agents such as chemotherapeutic agents, isoflavones have shown zero toxicity to humans ( Pop *et al.*, 2008).

**Figure 2.**
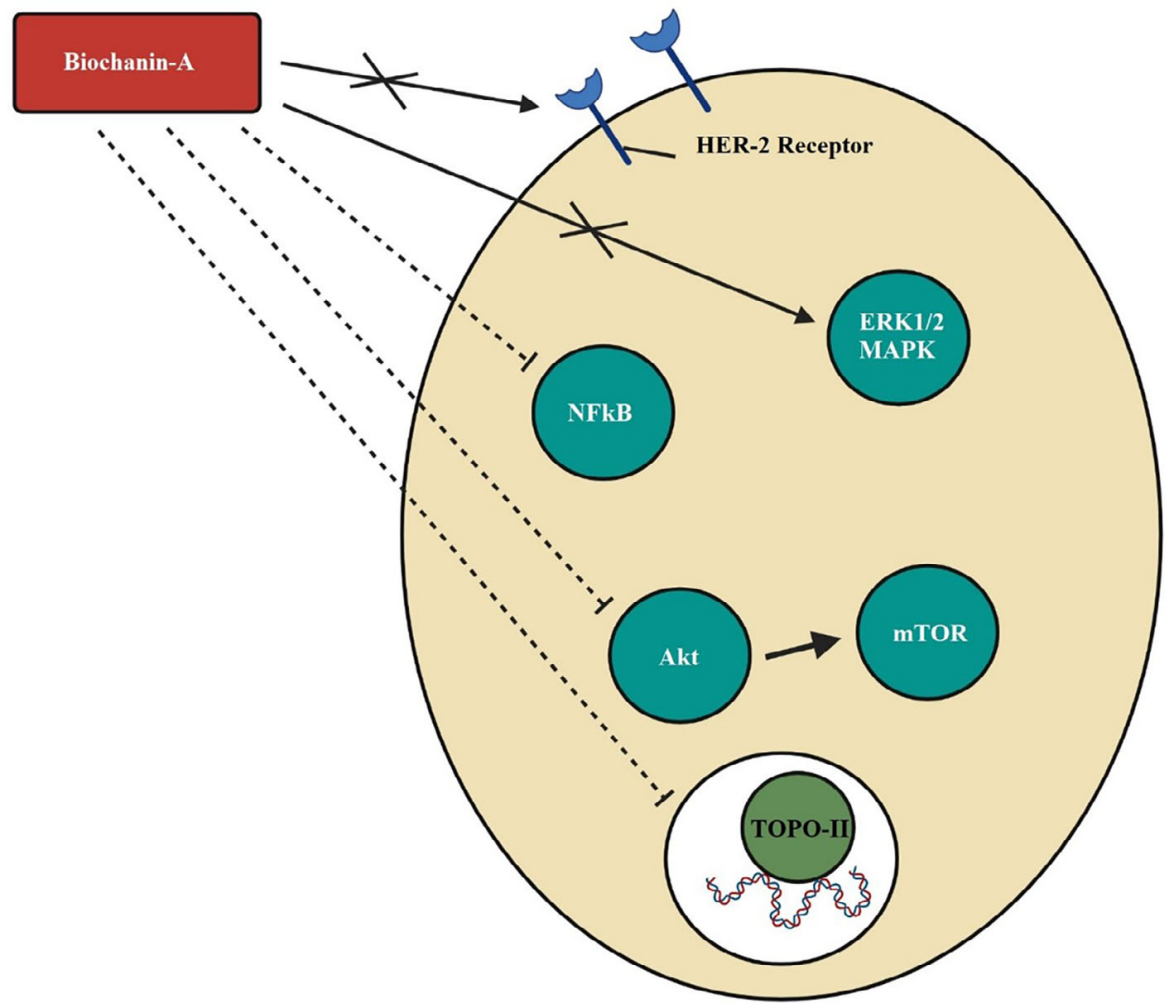
Mechanism of action of biochanin-A in breast cancer. Biochanin-A dephosphorylates HER-2 receptor and MAPK or ERK1/2 causing blockade of cancer cell development, growth, metastasis, and mitogenesis. Biochanin-A inhibits Akt phosphorylation thereby downregulates mTOR signals and disrupts the cell cycle. It inhibits NFkB and interrupts transcription. It inhibits topoisomerase-ll and DNA replication ( Azuma *et al.*, 1995; Gotoh *et al.*, 1998; Sehdev, Lai and Bhushan, 2009; Bhardwaj *et al.*, 2014).

With the influence of biochanin-A, HER2 receptor activation is inhibited, resulting in blockade of downstream signalling pathways of cancer cell development, viability, and metastasis. In HER2-positive breast cancer, the transcriptional unit nuclear factor (NF)-κB is suppressed ( Sehdev, Lai and Bhushan, 2009). MAPK or ERK 1/2 phosphorylation is inhibited causing the poor mitogenic effect ( Sehdev, Lai and Bhushan, 2009; Bhardwaj *et al.*, 2014). The major downstream signalling pathway Akt is dephosphorylated, consequently down-regulates the mTOR signalling pathway which regulates the cell cycle in SK-BR-3 breast cancer cells ( Sehdev, Lai and Bhushan, 2009). MMP-9 enzyme which facilitates metastasis of cancer cells using the extracellular matrix is repressed in SK-BR-3 cells treated with biochanin-A ( Sehdev, Lai and Bhushan, 2009). Flavonoids, having structural similarity to oestrogens, enable oestrogen receptor binding, and possess anti-oestrogenic and oestrogenic properties. Phytoestrogen inhibits oestrogen alpha receptors hence effective in oestrogen receptor-based treatment for breast cancer ( Collins, McLachlan and Arnold, 1997; Le Bail *et al.*, 1998). Reducing endogenous oestrogen levels in the body by inhibition of enzymes such as HSD and Cyp19 would defend against breast cancer. Phytoestrogen intake decreases oestrogen biosynthesis and prolongs menstrual cycle length thereby decreases lifetime exposure to oestrogen ( Mense *et al.*, 2008). Biochanin-A which is an AhR activator act as a cell cycle apoptotic stimulator, inhibiting DMBA (7,12 Dimethylbenz [a]anthracene) which can implicate in hormone-dependent cancer therapy and prevention ( Han, Ji and Hye, 2006; Medjakovic and Jungbauer, 2008). Biochanin-A inhibits CYP19 and negatively affects the synthesis of oestrogen in the body which enhances the anti-oestrogenic property in hormone-influenced cancer such as prostate cancer and breast cancer ( Mense *et al.*, 2008). Biochanin-A when combined with genistein and daidzein, significantly reduced Cyp19 enzyme activity and eliminated transcription of Cyp19 mRNA ( Rice, Mason and Whitehead, 2006). Biochanin-A blocks the cell proliferation in ER+ve MCF7 breast cancer cells ( Collins, McLachlan and Arnold, 1997). Oestradiol inhibition of biochanin-A at half maximal inhibitory concentration (IC _50_) dose is found with the highest inhibitory effect to 3-galactosidase action. The anti-oestrogenic activity of biochanin-A follows a mechanism analogous to tamoxifen ( Collins, McLachlan and Arnold, 1997). Topoisomerase II inhibition affects DNA replication. Biochanin-A through Topoisomerase II inhibition prevented the mammary tumor growth in N-nitro *-*N-methyl urea treated rat, interleukin 2-dependent CTLL-2 cells ( Azuma *et al.*, 1995; Gotoh *et al.*, 1998). Biochanin-A has shown synergism with 5-fluorouracil (5FU) in oestrogen receptor (ER) positive breast cancer cell lines such as MCF7, and triple-negative breast cancer cells such as MDA-MB231. The combination of 5FU and biochanin-A producing a synergistic anti-tumour effect partly attributed to the inhibitory capability of biochanin-A through ER-α/Akt ( Mahmoud *et al.*, 2022). The mechanism of action of biochanin-A in breast cancer by dephosphorylation of HER-2 receptor and MAPK or ERK1/2 causing blockade of cancer cell development, growth, metastasis, and mitogenesis, the inhibition of Akt phosphorylation and downregulation of mTOR signals that disrupt the cell cycle, the inhibition of NF-kB and interrupted transcription and blockade of topoisomerase-II and DNA replication is depicted in Figure 2.

**2.1.2 Prostate cancer**

Prostate cancer has been reported as a commonly found cancer in men and the second prominent death reason in western countries ( Siegel, Miller and Jemal, 2020). Diet can influence the prostate carcinogenesis process ( Bostwick *et al.*, 2004). Isoflavone contained dietary intake had presented with an association of reduced prostate cancer risk in different countries ( Jacobsen, Knutsen and Fraser, 1998; Applegate *et al.*, 2018).

Biochanin-A elevates the level of testosterone-UDPGT (Uridine 5′-diphospho-glucuronosyltransferase) enzyme activity and disrupts the androgen metabolism in connection with UDP-glucuronic acid. PLK-1 (Polo-like kinase-1) is responsible for various cell cycles activities such as Cdc2 (Cyclin-dependent kinase) stimulation and mitosis. Biochanin-A induces p21 which is a negative regulator for PLK-1 leading to prostate cancer cell apoptosis ( Seo *et al.*, 2011). The EGF (epidermal growth factor)-stimulated growth of cell lines, such as DU-145 and LNCaP prostatic cancer, were inhibited by biochanin-A without affecting its autophosphorylation. Biochanin-A has shown inhibitory behaviour in prostate cancer by antagonizing tyrosine kinase events within the signal transduction pathway ( Peterson and Barnes, 1993). The level of testosterone and development of prostate carcinoma in Lobund-Wistar rats were influenced through a soy-rich diet, signifying preventive action of soy isoflavones in prostate cancer ( Pollard, Wolter and Sun, 2000). Using the orthotopic prostate tumour animal model, the influence of soy proteins on cancer advancement has been studied. PSA androgen sensitivity, and cancer metastasis was inhibited significantly by different soy-derived compounds ( Zhou *et al.*, 2002). ER-β, when activated in prostate cells, inhibits cell proliferation, and exerts anti-cancer effects. Red clover supplemented diet in mice exhibited increased ER-β and E-cadherin levels leading to the disruption of cell morphology and cancer formation ( Slater, Brown and Husband, 2002). Biochanin-A in a LNCaP cell line induced apoptosis incorporated by 3H-thymidine with increased DNA fragmentation, low p21, and cyclin B expression. Animal study with LNCaP xenografts, biochanin-A subsided the prostate cancer load and size of the tumour ( Rice *et al.*, 2002). The prostatic androgen 5α-dihydrotestosterone synthesized using a 5α-reductase enzyme which is responsible for prostate cell development and function has been influenced by biochanin-A, thus generate a role in the prevention of prostate malignancy ( Evans, Griffiths and Morton, 1995).

Phytoestrogens have been predicted as compounds liable for chemoprotective activity on prolonged exposure. Prostatic cell proliferation in PC-3, LNCaP, and DU145 were inhibited by biochanin-A with variable mechanisms ( Hempstock, Kavanagh and George, 1998). Aromatase enzyme has an impact on the level of oestrogen. Biochanin-A upon competitive inhibitory action on aromatase minimizes oestrogen level and exhibit anti-cancer activity ( Campbell and Kurzer, 1993). TRAIL-induced cell death is an epitome in cancer prevention. Biochanin-A through deactivating NF-kB and death receptor (DR) 4/5 mediated caspases causes TRAIL-associated cell death in prostatic cancer cell lines ( Szliszka *et al.*, 2013). Increased activity of UDP-glucuronosyltransferase (UDGPT) is found in biochanin-A-exposed LNCaP cells. It enhanced intracellular glucuronidation of testosterone, steroid UDGPT transcript, and lowered prostate-specific antigen (PSA), hence showed an effect in prostate cancer prevention ( Sun *et al.*, 1998). The patients with clinically significant prostate cancer were treated with red clover isoflavones such as biochanin-A before surgical intervention. The apoptosis markers in prostate tumor cells from radical prostatectomy specimens were analysed. Markedly, higher apoptosis was found in the treatment group with a dietary supplement of isoflavones, indicating cessation of prostate cancer progression in low to moderate-grade malignancy ( Jarred *et al.*, 2002).

**Figure 3.**
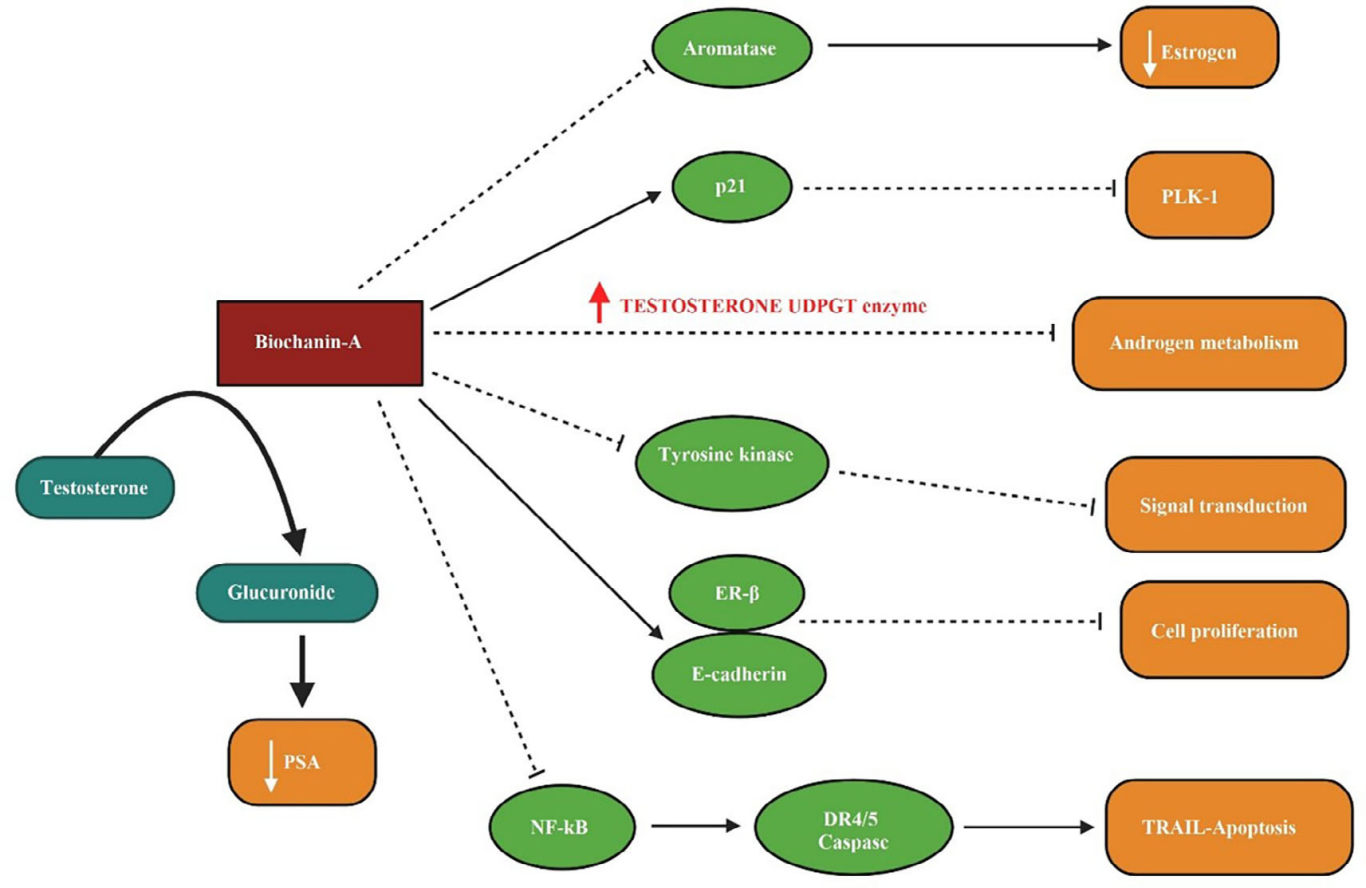
Biochanin-A on prostate cancer. Biochanin-A inhibits aromatase enzyme and decreases estrogen levels. It activates p21 and antagonizes PLK-1 action. Increased level of testosterone UDGPT enzyme disrupts androgen metabolism with the treatment of biochanin-A. The level of tyrosine kinase is interrupted and inhibits signal transduction. It induces ER-β and E-cadherin and inhibits cell proliferation. Biochanin-A inhibits NF-kB and induced TRAIL associated apoptosis. It increased conversion of testosterone into glucuronide resulting low appearance of prostate-specific antigen (PSA) ( Sun *et al.*, 1998; Seo *et al.*, 2011; Szliszka *et al.*, 2013).

In Figure 3 the inhibition of aromatase enzyme and lowering of oestrogen level by biochanin-A, the activation of p21 and antagonization of PLK-1 action, increased level of testosterone UDGPT enzyme disrupting androgen metabolism with the treatment of biochanin-A, the interrupted level of tyrosine kinase blocking the signal transduction, the prompted ER- β and E-cadherin level leading to the inhibition of cell proliferation, via biochanin-A inhibition of NF-kB inducing the TRAIL associated apoptosis and the increased conversion of testosterone into glucuronide resulting low appearance of prostate-specific antigen (PSA) is illustrated.

**2.1.3 Lung cancer**

Cancer affecting the most vital body parts elucidate the difficulty of its therapy. Lung cancer has often been reported and is one of the supreme death causes in cancer patients. The fight against cancer with phytochemicals will benefit mankind greatly. Studies have been carried out on soy isoflavones, utilized as an integral system for the treatment and to boost the radiation viability on lung tumors ( Hillman and Singh-Gupta, 2011; Singh-Gupta *et al.*, 2011). Soy-rich foods may reduce the occurrence of cancer development in the general population, according to epidemiology research ( Yang *et al.*, 2011). Like genistein which has proven its effectiveness in cancer, biochanin-A displays anti-cancer properties in lung tissue.

The mechanism with which soy isoflavones boost radiation therapeutic effect is through inhibiting APE1/Ref-1 DNA repair in A549 cells, which leads to cell killing ( Singh-Gupta *et al.*, 2011). Soy isoflavones exhibited a synergistic effect and significantly improved the radiation-induced cell killing. in vitro observation on biochanin-A treatment in 95D and A549 lung cancer cells revealed that the level of P21 (cyclin dependent kinase-1), Caspase-3, and Bcl-2 were stimulated causing cell cycle arrest and death. Dose-proportional apoptosis and prevention of DNA replication in the S phase by biochanin-A were observed. A good level of caspase-3 and reduced Bcl-2/Bax proportion facilitates apoptosis and lung cancer prevention ( Li *et al.*, 2018). Biochanin-A elicited pro-inflammatory properties beneficial to anti-cancer effect in lung cancer. AML 193 and A427 were tested with exposure of biochanin-A, the release of IL-6 cytokines and TNF-α, low level of E cadherin, and Snail blocking to epithelial–mesenchymal transition (EMT) which is essential in tumour growth and metastasis ( Wang, Li and Chen, 2018). In an in vivo study with benzo(a)pyrine-induced lung cancer, the biochanin-A treated group had displayed a marked decrease in the development of tumour at a dose less than dose causing a 10% lethality (LD10) ( Lee *et al.*, 1991). When soy and red clover extracts were tested in NSCLC in combination with anti-cancer agents like gefitinib, erlotinib, afatinib, and osimertinib, the results were satisfactory. Particularly, the red clover extract which essentially contains biochanin-A showed a synergic effect with EGFR inhibitors and significant inhibition of tumor growth ( Ambrosio *et al.*, 2016).

**Figure 4.**
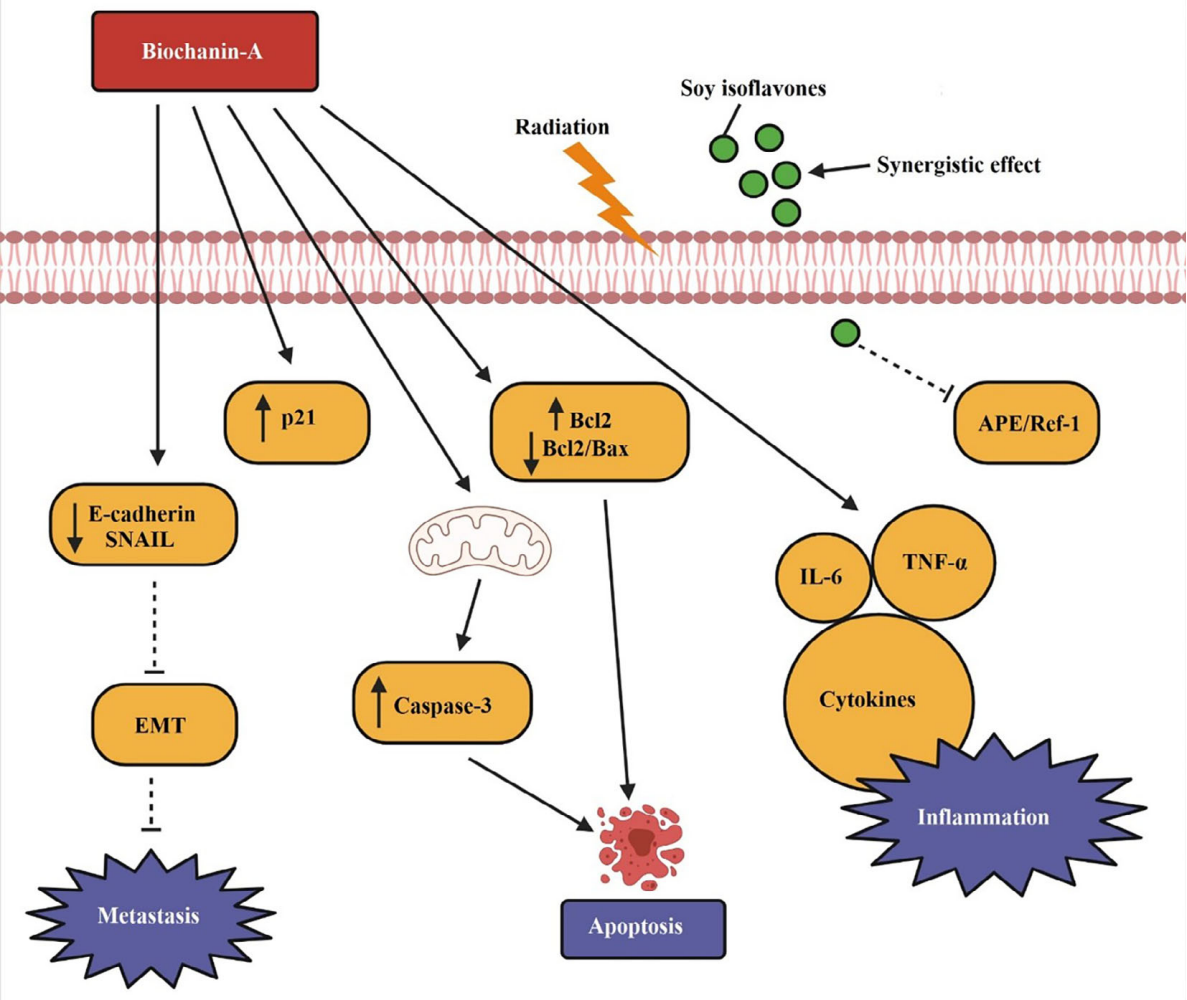
Apoptotic pathway by biochanin-A on lung cancer. Biochanin-A cause apoptosis in lung cancer by increasing p21, caspase-3, and Bcl-2 levels. It lowers E-cadherin and blocks metastasis. Pro-inflammatory mediators such as TNF-α, IL-6, and cytokines are released to facilitate apoptosis. Soy isoflavones along with radiation therapy shows a synergistic effect in causing cell death by inhibiting APE/Ref-1 ( Singh-Gupta *et al.*, 2011; Li *et al.*, 2018; Wang, Li and Chen, 2018).

Figure 4 demonstrates the apoptotic pathway by biochanin-A on lung cancer. p21, caspase-3, and Bcl-2 levels are elevated by biochanin-A, which causes lung cancer to undergo apoptosis. Both metastasis and E-cadherin are reduced. TNF-a, IL-6, and cytokines are only a few examples of the pro-inflammatory mediators that are generated to aid in apoptosis. Soy isoflavones along with radiation therapy shows a synergistic effect in causing cell death by inhibiting APE/Ref-1 as depicted in the figure.

**2.1.4 Pancreatic cancer**

The low survival rate of pancreatic cancer with limited anti-cancer agents makes it difficult to manage the disease. It is also known to be the most aggressive one among other cancers ( Baghurst *et al.*, 1991; Moore *et al.*, 2003). Mutations in the tumour-suppressor and tumour-promoting gene are attributed to the aggressiveness of the disease ( Saif, 2007). Association with high-calorie diet and increased incidence of pancreatic cancer are reported ( Lowenfels and Maisonneuve, 2005; Li, 2009; Arslan *et al.*, 2010; Chang *et al.*, 2017). Consequently, it is important to conduct research on the role of isoflavones in pancreatic cancer ( Silverman *et al.*, 1998). Biochanin-A had a negative influence on pancreatic cancer progression with variable mechanisms.

The cluster formation ability of pancreatic cancer cells Panc1 was hindered by biochanin-A with dose-dependent toxicity. It inhibited mitosis, migration, and invasion of pancreatic cancer progression. EGFR, Akt, and MAPK pathways are deactivated resulting in apoptosis in Panc1 and AsPC-1 cell lines suggesting combination therapy with biochanin-A could be considered for treatment ( Bhardwaj *et al.*, 2014). Biochanin-A along with atorvastatin enhanced anti-cancer properties on AsPC1, MIAPaCa-2, and PANC-1 cell lines by lowering cell invasiveness and cell cycle progression. Biochanin-A interferes with cell survival by decreasing MAPK and Akt hence affects mitogenic signalling ( Desai *et al.*, 2018). Concentration-dependent cellular invasiveness and migration are found with biochanin-A by reducing the level of matrix metalloproteases (MMP) indicating pancreatic cancer cell migration is inhibited ( Bhardwaj *et al.*, 2014).

**2.1.5 Colon cancer**

It is the most prevalent type of cancer in existence today. Statistics by the American Cancer Society, 2019, shows that colon cancer is the second leading source of mortality in cancer. As specified by American Institute for Cancer Research and World Cancer Research Fund reports dietary factors may elucidate the risk of having colorectal cancer. Intake of isoflavone-contained food like soy influences gastric cancer occurrence ( Yan, Spitznagel and Bosland, 2010; Ko *et al.*, 2013).

The synergism of biochanin-A with 5-fluorouracil evidenced in Caco-2 and HCT-116 cell lines indicates the modulatory influence of biochanin-A in colon cancer treatment. The biochanin-A on its own shows cytotoxicity in the cell lines. It blocked the “Akt and GSK3β phosphorylation and boosted the degradation of β-catenin” ( Mahmoud *et al.*, 2017). Biochanin-A when combined with gamma radiation on HT29 cells, which is resistant to radiation, had revealed a reduction in cell proliferation. Raised levels of ROS, lipid peroxidation, MMP, caspase-3 have been observed more in the treatment group with significant apoptosis ( Puthli, Tiwari and Mishra, 2013). Biochanin-A and other isoflavones displayed a growth-retarded effect on HCT-116/SW-480 in a time and dose-reliant manner ( ZHANG *et al.*, 2013). Stomach cancer cell lines SH101-P4, HSC-45M2, HSC-41E6, and colon cancer cell lines have been treated with isoflavones including biochanin-A and observed cytostatic effect. DNA fragmentation, chromatin condensation, and nuclear fragmentation of each cell line are seen with the apoptotic result ( Yanagihara *et al.*, 1993). Oestrogen sensitive cancer cell lines including colon cancer cell 320DM when treated with biochanin-A and other isoflavones revealed antiproliferative effect which is beneficial as a cancer preventative ( Kohen *et al.*, 2007).

**Figure 5.**
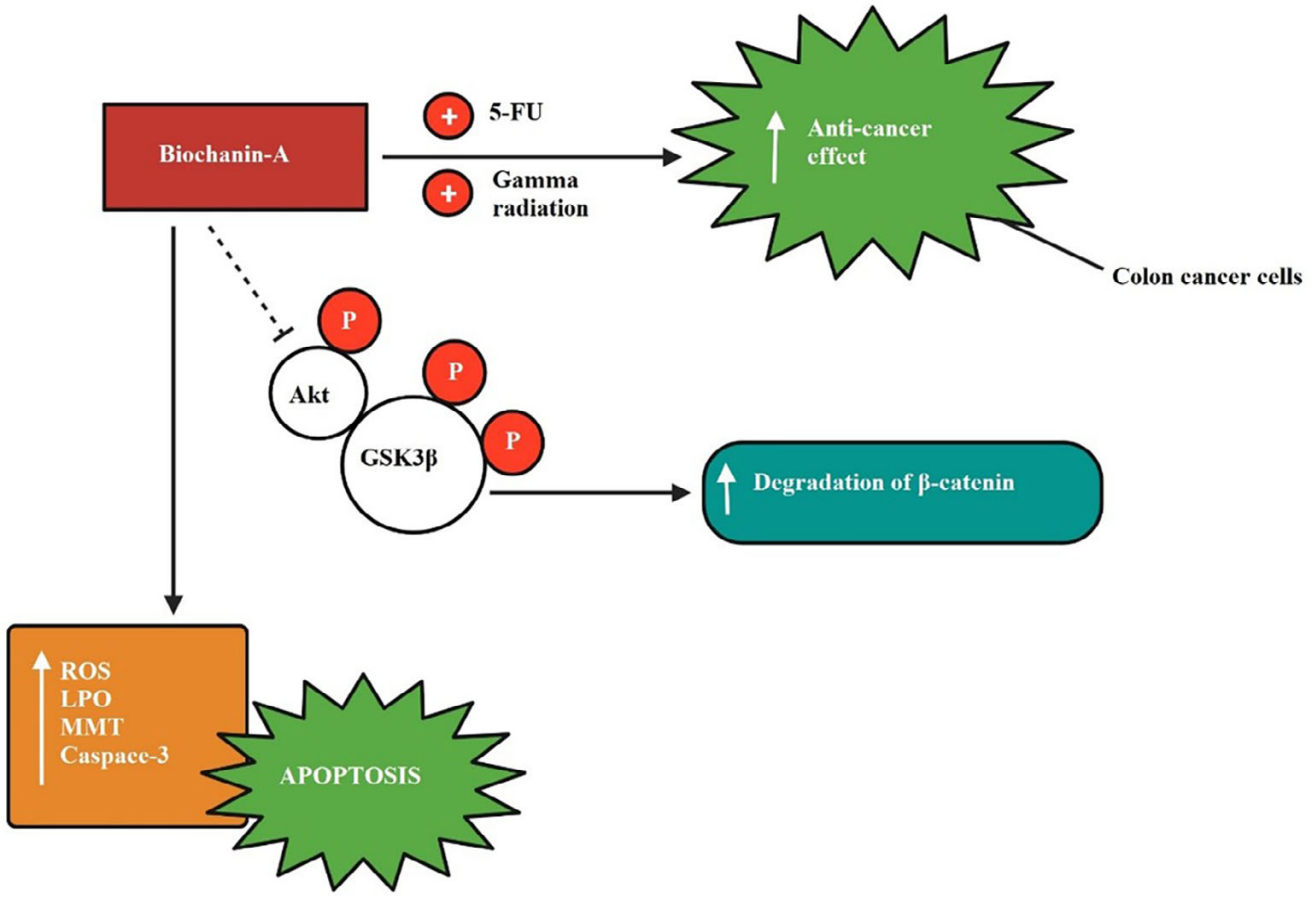
Colon cancer inhibition by biochanin-A. Biochanin-A boosted the anti-cancer effect exerted by 5-fluorouracil and gamma radiation when given in combination in colon cancer cells. Biochanin-A has inhibited Akt and GSK3β phosphorylation ( Puthli, Tiwari and Mishra, 2013; Mahmoud *et al.*, 2017).

The inhibitory mechanism of biochanin-A on colon cancer is shown in Figure 5 explaining biochanin-A when given in combination enhanced the anti-cancer effect exerted by 5-fluorouracil and gamma radiation in colon cancer cells. Biochanin-A has inhibited Akt and GSK3β phosphorylation.

**2.1.6 Glioma**

Glioblastoma multiforme (GBM), the most widely reported and destructive brain malignancy, has a high death rate. GBM tends to reappear since it displays both intra- and inter-tumoral heterogeneity ( Shergalis *et al.*, 2018). The therapy becomes especially challenging due to the existence of BBB and rapid penetration to neighbouring tissue ( Desai and Bhushan, 2017). The chemo preventive and anti-angiogenic properties of isoflavones along with refining the adequacy of chemotherapy and radiotherapy for the treatment of GBM have received much attention lately ( Tedeschi-Blok *et al.*, 2006; Sarkar *et al.*, 2009).

In a dose-dependent manner, biochanin-A influenced the tumour invasion capacity by lowering matrix-degrading enzymes (MMP 2 and MMP 9) tested in U87MG cells ( Puli, Lai and Bhushan, 2006). Biochanin-A inhibited endothelial cell functions in rat brain tumour, brain endothelial cells, and chick chorioallantoic membrane model with its anti-angiogenic properties through ERK/AKT ex vivo/mTOR dephosphorylation ( Jain, Lai and Bhushan, 2015). Biochanin-A along with temozolomide disclosed exceptional anti-cancer activities in human glioblastoma cells, U87 MG, and T98-G. Biochanin-A by lowering EGFR, p-ERK (Extracellular signal related kinases), p-AKT (Protein kinase-B), c-myc, and MT-MMP1 (Membrane type matrix metalloproteinase) activation, inhibited cell survival. It influenced the abilities of cancer cells in viability, DNA repair, proliferation, and cell cycle arrest. Biochanin-A synergistically improved temozolomide anti-cancer ability in GBM ( Desai *et al.*, 2019). Biochanin-A augments temozolomide by lowering the number of colonies and p-EGFR, p-ERK, uPAR (Urokinase type plasminogen activator receptor), MMP-2 levels in GBM cells ( Jain, Lai and Bhushan, 2011). Biochanin-A has proven to be a better candidate in GBM management in comparison with other isoflavones. It is found that biochanin-A exhibits a protective effect in a multimodal treatment methodology via testing in glioma cells (in-vitro), IP injection to tumour-implanted Fisher rats (in-vivo), and organotypic brain slices as ex-vivo experiments ( Sehm *et al.*, 2014). Cell signalling pathways MAP kinase, PI3 kinase, mTOR, matrix metalloproteases, hypoxia-inducible factor, and VEGF were inhibited by biochanin-A, making it suitable in treating GBM ( Bhushan, Jain and Lai, 2015). In glioma C6 cells, the activation of ERK/Akt, the pro-angiogenic proteins were blocked by biochanin-A, and also VEGF and HIF-1α (hypoxia-inducible factor 1 alpha) were inhibited ( Jain, Lai and Bhushan, 2015). While testing the effect of rapamycin combined with biochanin-A in U87 glioma cells, there was decrease in cancer invasion and matrix-degrading enzymes. The combination dephosphorylated Akt and eIF4E (Eukaryotic translation initiation factor) augmenting rapamycin drug effect ( Puli, 2006). In individuals with glioblastoma, tolerance to temozolomide (TMZ) chemotherapy is the most common cause of the relapse of GBM. Biochanin-A was found to be a strong TMZ sensitizer in GBM, by increasing the cell sensitivity through AMPK/ULK-1 pathway ( Dong *et al.*, 2022).

**2.1.7 Osteosarcoma**

Osteosarcoma (OS), a malignant bone tumour, is generally seen in children and adolescents. The low survival rate of OS is associated with drug resistance causing the poor response to chemotherapy. Hence, phytochemicals that can contribute to OS treatment are significant.

Biochanin-A and doxorubicin together suppressed the tumour development by promoting the release of apoptotic factors, damaging mitochondrial membrane potential, eliciting “the intrinsic mitochondrial pathway, caspase-9 and -3 activation” and increasing “Bax: Bcl-2/Bcl-XL ratio” ( Hsu *et al.*, 2018). MG63 and U2OS osteosarcoma cells treated with biochanin-A revealed cytotoxicity at the molecular level. It is an apoptosis inducer (caspase-3), cell proliferation and invasion inhibitor, and dephosphorylates PCNA (proliferating cell nuclear antigen) and cyclin D1 gene expression ( Zhao *et al.*, 2018b). The expression of caspase-3 controlled by biochanin-A while regulating cell death is one possible mechanism to manage osteosarcoma. With the molecular docking technique it was found that certain proteins were identified as effective targets of biochanin-A for osteosarcoma. Among those “BGLAP, BAX and ATF3” were recognized as the potential target of interest in blocking cancer cell proliferation using biochanin-A ( Luo *et al.*, 2019). Biochanin-A tested in MG63 and U2OS cell lines exhibited a time and dose-related inhibitory effect on cancer proliferation, cell death, infiltration, and metastasis ( Zhao *et al.*, 2018b).

**2.1.8 Leukaemia & other cancers**

Leukaemia or blood cancer affects the most important connective tissue of our body: blood, and the blood-forming tissue. It is considered a deadly and serious form of cancer ( Leukaemia Care, 2020). However, isoflavones have revealed their protective role in several investigations ( Xu *et al.*, 2008). Soy derivative isoflavones obstruct the cell cycle of leukemic cells ( Yamasaki *et al.*, 2007).

Biochanin-A in JCS cells prompted monocytic differentiation to macrophages (markers “Mac-l and F4/80”) showing increased phagocytic activity. The cytokines production (“IL-la, IL-lo, IL-4 and TNF-α”) is regulated in the late stage of monocytic differentiation of JCS cells by biochanin-A ( Fung *et al.*, 1997). Biochanin-A affects intracellular antioxidant response system via Nrf2-Anti oxidant response element signalling pathway in tert-butyl hydroperoxide (t-BHP)-induced oxidative damage in HepG2 cell line. Biochanin-A binds to Keap1’s pocket, causing Nrf2 signalling to be activated. These results suggest that dietary isoflavones may protect liver cancer patients from oxidative damage ( Liang *et al.*, 2019). A study has found that Biochanin-A can be used to treat multiple myeloma, which is a type of cancer that affects plasma B cells in the bone marrow. The application of Biochanin-A has been shown to reduce the level of CD38, which is one of the key therapeutic targets for combating Multiple Myeloma. It has also been found to trigger apoptosis in Multiple Myeloma cells and reduce cytokine expression. The study further explored NOD/SCID mice with U266-induced tumors, Biochanin-A treatment was found to significantly reduce tumour growth. Mechanistic studies have shown that Biochanin-A’s anti-cancer effects are achieved by modulating the NF-κB and MAPK signalling pathways. This study suggests that Biochanin-A may offer a new, more effective, and less toxic type of treatment for multiple myeloma ( Jaina *et al.*, 2022).

Anti-cancer effects of biochanin-A substantiated through in vivo and in vitro experiments is summarized in Table 1.

**Table 1.** Anti-cancer effects of biochanin-A substantiated through in-vivo and in-vitro experiments is summarized in Table 1. ( **↑**increase, ↓decrease or × inhibit, + activation).

Pharmacological Application	Study models	Dose used	Major findings\mechanisms	Reference
Breast cancer	HER-2 Positive breast cancer cell lines	2–100 μM	NFκB ↓ Unique anticancer agent MAPK or ERK 1/2 phosphorylation ×	( Sehdev, Lai and Bhushan, 2009)
	MCF-7 human breast carcinoma cells	10–50 μM	Cell cycle apoptotis + DMBA ×	( Han *et al.*, 2006)
	Human granular luteal cells	10–100 nm	Cyp19 enzyme ↓ Have role in aromatase activity	( Rice, Mason and Whitehead, 2006)
	MCF-7 breast cancer cell lines	500 nm	Anti-oestrogenic property	( Collins, McLachlan and Arnold, 1997)
	CTLL-2 cells (Murine IL-2 dependent T cell clone	5–50 μM	Topoisomerase II ×	( Azuma *et al.*, 1995)
Prostate cancer	LNCaP cell lines	0.5–50 μM	testosterone-UDPGT ↑ PSA ↓ Androgen metabolism ×	( Sun *et al.*, 1998)
	PC-3 (p53 mutant) and LNCaP (p53 wild type)	100 μM	p21 ↑ PLK-1 ↓ Cell apoptosis +	( Seo *et al.*, 2011)
	DU-145 and LNCaP	8.0 μg/mL for LNCaP 9.0 μg/mL for DU-145 cells	EGF-stimulated growth × tyrosine kinase events ×	( Peterson and Barnes, 1993)
	Red clover diet Model (Isoflavones constitute 1.26% of the diet)	Biochanin-A 5.74 mg isoflavone/g pellet	ER-β and E-cadherin levels ↑ Cancer formation ×	( Slater, Brown and Husband, 2002)
	LNCaP cells Athymic mice with LNCaP flank tumors	10 μg/mL 400 μg for animal xenographs model	DNA fragmentation ↑ p21 and cyclin B ↓ Tumor size and incidence ↓	( Rice *et al.*, 2002)
	Fibroblast cell	100 μg/mL	5 α-reductase isozymes × hormone-dependent tumours ↓	( Evans, Griffiths and Morton, 1995)
	LNCaP cells and DU145 cells,	20–100 μM	TRAIL-associated cell death NF-κB ×	( Szliszka *et al.*, 2013)
	PC-3, LnCaP, and DU145	100 μmol/L	Cell proliferation × Growth and metabolism ×	( Hempstock, Kavanagh and George, 1998)
Lung cancer	95D and A549 cancer cells injected to male nude mice	Biochanin-A IP 15,60 mg/kg group of A549 18,75 mg/kg group of 95D	Cell proliferation of lung cancer cells ×	( Li *et al.*, 2018).
	Human lung adenocarcinoma cell line(A427),Human monocyte leukaemia cell line (AML-193)	5, 20, 40 μM	Hinderes proinflammatory effects triggered from leukaemia	( Wang *et al.*, 2018
	New born mouse model	65 mg/kg	Carcinogenesis ×	( Lee *et al.*, 1991)
	Non-small cell lung cancer	Soy red clover isoflavones	Isoflavones combined with EGFR inhibitors improve NSCLC cell growth	( Ambrosio *et al.*, 2016)
Pancreatic cancer	Panc/AsPC-1	5 mg/mL	Cell proliferation × Apoptosis +	( Bhardwaj *et al.*, 2014)
Colon cancer	Caco-2,HCT-116 cell lines	34 μM	Biochanin-A combination with 5 FU promotes management of colon cancer	( Mahmoud *et al.*, 2017)
	HT29cells	1–100 μM	Biochanin-A increases radiotoxicity	( Puthli, Tiwari and Mishra, 2013)
	HCT-116 Sw-480 cell lines	2.5–100 μM	Biochanin-A enhances genotoxYangiharaic effects and antitumor mechanism	Yanagihara *et al.*, 1993)
	320 DM cell line	20 μM	Biochanin-A is a promising target for cancer	( Kohen *et al.*, 2007)
Glioblastoma multiforme	U87MG	50 μM	MMP 2 and MMP 9 ↓	( Puli, Lai and Bhushan, 2006)
	Rat brain tumour (C6) Murine brain endothelial (bEnd.3) cells Ex-vivo chick chorioallantoic membrane model	5, 35, and 70 μmol/L	Anti-angiogenic properties through ERK/AKT/mTOR dephosphorylation	( Jain, Lai and Bhushan, 2015)
	U-87 MG, and T98 G	70 μM	EGFR, p-ERK, p-AKT, c-myc, and MT-MMP1 activation ↓ cell survival * Synergism to anti-cancer ability of Temozolomide	( Desai *et al.*, 2019)
	U-87 human glioblastoma cell line	20 μM and 70 μM	p-EGFR, p-ERK, uPAR, MMP-2 ↓	( Jain, Lai and Bhushan, 2011)
Osteosarcoma	MG63 and U2OS Cells	20±0.3 μg/mL	Bax: Bcl-2/Bcl-XL ratio ↑ caspase 9 & 3 + MMP ↓	( Hsu *et al.*, 2018)
	MG63 and U2OS Cells	40 μM	Apoptosis + caspase-3 ↑ Cell proliferation and invasion ×	( Zhao *et al.*, 2018b)
Leukaemia	JCS		Monocytic differentiation to macrophages + Phagocytic activity ↑	( Fung *et al.*, 1997)

### 2.2 Metabolic disorders

**2.2.1 Diabetes**

Diabetes mellitus, an age-old metabolic disorder, has a high rate of occurrence around the world. It is described as “hyperglycaemia” caused by deformities in insulin secretion, insulin activity, or both ( American Diabetes Association, 2009). The long-term presence of diabetes is associated with many other complications. Even though currently, accessible medications might be significant in the control of diabetes, these medications are joined with certain side effects as well. A few varieties of phytochemicals have shown potential for the management of diabetes with minor or no side effects ( S.Mohana Lakshmi, Rani and Reddy, 2012; Surya *et al.*, 2014). Bioflavonoids are remarkable for their hypoglycaemic abilities ( Vinayagam and Xu, 2015). It has been exhibited that flavonoids can go about as “insulin secretagogue or insulin-mimetic agents” ( Patel *et al.*, 2012; Singh and Sahu, 2018).

Biochanin-A action in streptozotocin-induced diabetic rats displayed improved glucose digestion and dropped HbA1C levels. Serum visfatin amount was enhanced ( Azizi, Goodarzi and Salemi, 2014). STZ diabetic rats on oral treatment with biochanin-A exposed anti-diabetic properties by lowering FBS and hyperglycaemia-induced free radicals ( Sadri *et al.*, 2017). Red clover extract was tested in “db/db diabetic mice” to see the anti-diabetic and anti-hyperlipidemic activities. Increased hepatic PPARα/γ stimulation and reduced hepatic fatty acid synthase levels contributed to achieving glucose and lipid homeostasis by red clover compounds ( Qiu, Ye, *et al.*, 2012b). STZ-diabetic C57BL/6 mice were treated with red clover extracts including biochanin-A and formononetin. The lipid profile of the animal was influenced by biochanin-A rather than glucose levels through mechanisms in connection with hepatic PPARa ( Qiu, Ye, *et al.*, 2012a). The raised levels of plasma glucose, HbA1C, and gained weight were normalized with biochanin-A treatment ( Harini, Ezhumalai and Pugalendi, 2012). At certain doses, biochanin-A reduced glucose tolerance and insulin resistance, developed insulin sensitivity and increased SIRT-1 expression which might explain the anti-diabetic properties of the drug ( Oza and Kulkarni, 2018). Biochanin-A acts as a strong PPAR receptor activator (PPARalpha/PPARgamma) even at low doses signifying its anti-diabetic and anti-hyperlipidaemic properties ( Shen *et al.*, 2006). Nesfatin-1, a regulatory peptide level, and insulin were increased upon biochanin-A treatment in type-1 diabetic rats, which can hint to its one probable mechanism behind the hypoglycaemic property ( Eskandari Mehrabadi and Salemi, 2016). The structural relationship and activity of biochanin-A and other isoflavones were checked. Biochanin-A derivative (7-diethyl phosphite-O-biochanin-A) had shown greater anti-hyperglycaemic activity compared to all other compounds ( Wei *et al.*, 2017). The role of serum adiponectin and serum resistin as a glucose metabolism regulator in diabetes is discoursed and it is found that biochanin-A improved adiponectin release and augments insulin activity. The elevated resistin usually observed in the type-1 diabetic condition is decreased after biochanin-A intake. The oxidative stress produced in diabetes was also taken care of by biochanin-A ( Salemi *et al.*, 2018).

The influence of biochanin-A in diabetic neuropathy using an STZ-induced rat model revealed that, mechanical allodynia and hyperalgesia (paw withdrawal threshold) were reversed upon treatment. Hence, biochanin-A could be a drug of choice in diabetic neuropathy ( Chundi *et al.*, 2016). The retina of diabetic rats was tested to rule out diabetic retinopathy after biochanin-A treatment. It was uncovered that it significantly prolonged the event of retinal damage with its anti-inflammatory and anti-angiogenic property ( Mehrabadi *et al.*, 2018). Diabetic nephropathy caused due to increased oxidative stress and TGF-β, was monitored in type 2 diabetes mellitus-induced rats to know if biochanin-A can play a role. It had significantly improved kidney function through modulating TGF-β expression and minimizing oxidative stress ( Ahad, 2013). Hyperlipidaemia is a common comorbidity seen in diabetic patients. Biochanin-A administered to diabetic animals demonstrated low fasting blood sugar (FBS) as well as small dense low density lipoprotein cholesterol (sd-LDLC), favourable in diabetic dyslipidaemia conditions ( Ghadimi *et al.*, 2019). A formulation containing biochanin-A with or without its analogues will be beneficial in treating diabetes and diabetic cardiomyopathy, with evidence through increasing IGF1R (insulin-like growth factor 1 receptor), INSR (insulin receptor), and IRS2 (insulin receptor substrate 2) levels, thereby leading to the up-regulation of Lin28 gene and insulin sensitivity ( Boominathan L, 2017). Biochanin-A was administered for 16 weeks orally once in a day to HFD-fed rats with single dose streptozotocin and showed that it has the ability to increase SIRT1 expression in heart tissue while also controlling hyperglycaemia and oxidative stress. Biochanin-A could be a promising candidate for lowering the advancement of cardiomyopathy in people with type 2 diabetes ( Oza and Kulkarni, 2022). A study designed to examine the diabetic and diabetic nephropathy effects on diabetic rats revealed that administering biochanin-A markedly reduced the expression of transforming growth factor-β1 (TGF-β1), protease-activated receptors 2 (PAR-2) genes, and fasting blood glucose (FBG) ( Amri *et al.*, 2021).

**Figure 6.**
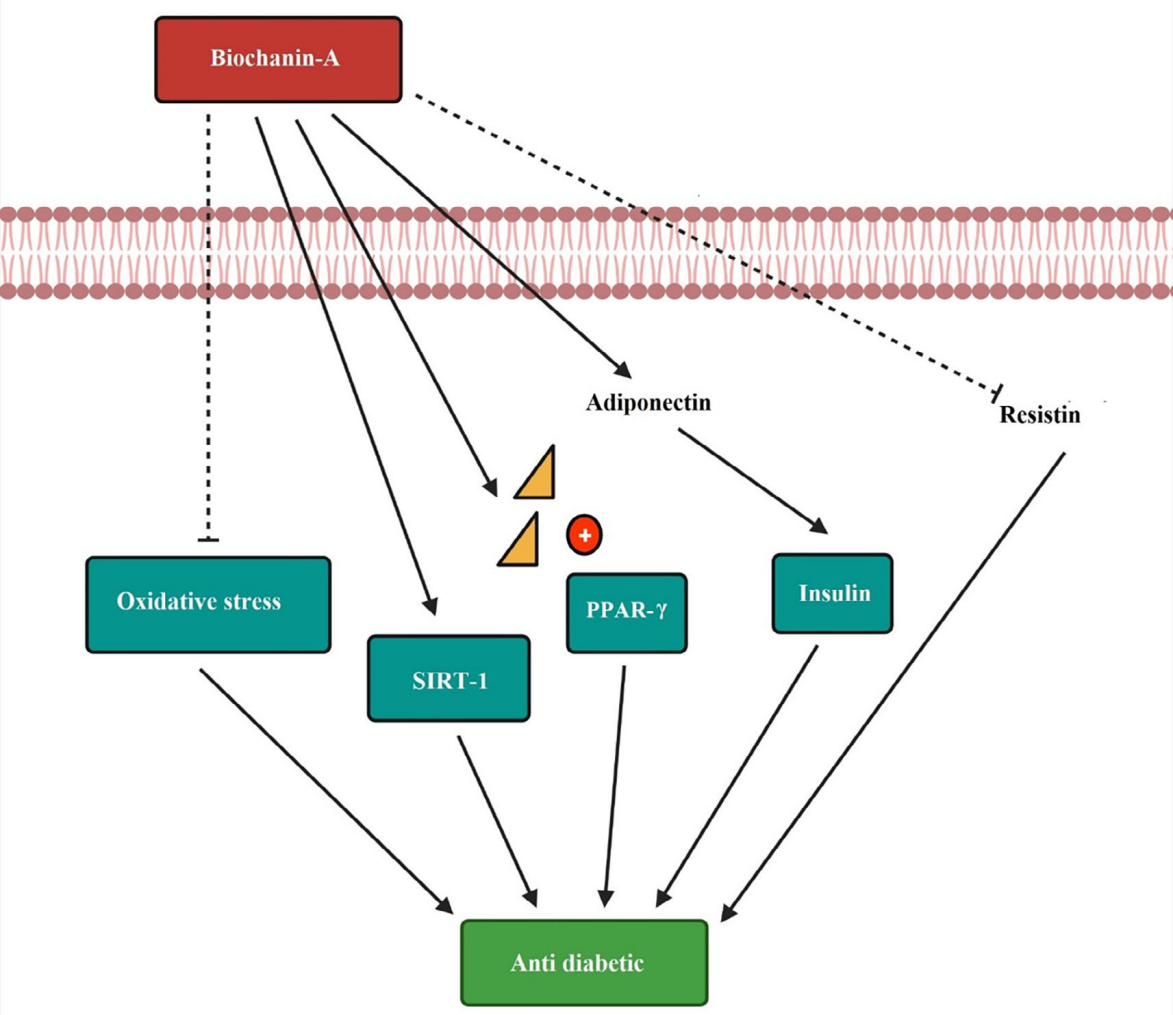
Anti-diabetic mechanism of biochanin-A. Biochanin-A decreases the oxidative stress and helps in diabetes condition. It increases the expression of SIRT-1 and progresses insulin sensitivity. Biochanin-A is a PPAR-γ receptor activator producing an anti-diabetic effect. It increases the release of adiponectin and decreases the resistin level to improve the diabetic condition ( Shen *et al.*, 2006; Oza and Kulkarni, 2018; Salemi *et al.*, 2018).

The anti-diabetic mechanism of biochanin-A is by decreasing oxidative stress. SIRT-1 influences the progression of insulin sensitivity. Biochanin-A act as a PPAR gamma receptor activator and produces anti-diabetic effect. The increased release of adiponectin and low resistin level to improve the diabetic condition is depicted in Figure 6.

**2.2.2 Dyslipidaemia**

Dyslipidaemia or hyperlipidaemia is a very common metabolic disorder characterized by a high level of triglycerides and low-density lipoproteins, responsible for cardiovascular diseases ( Thompson, 2004). The association between soy diet and hyperlipidaemia is a discussed topic among researchers. Soy can normalize the increased cholesterol level while taken in combination with conventional hyperlipidaemic drugs ( Costa and Summa, 2000). A randomized control trial on isoflavones in hypercholesterolaemia showed that there was a minor significant positive impact on triglycerides levels supporting this fact ( Qin *et al.*, 2013).

Treatment with a moderate dose of biochanin-A in HFD mice has shown a substantial lowering of LDL and total cholesterol. Lipoprotein lipase and hepatic triglyceride lipase levels are increased. Molecular docking studies on biochanin-A displayed a noteworthy role in reducing cholesterol-ester transport ( Xue *et al.*, 2017). Biochanin-A over formononetin reduced LDL cholesterol in men, though the same was not observed in women ( Nestel *et al.*, 2004). Biochanin-A lowers blood lipid levels, blood fibrinogen, and blood thickness levels of rats with hyperlipidaemia, increases blood circulation, and alters the blood coagulation system in lipid metabolism disorders ( Ling-hui *et al.*, 2012). Plant sterol combined with soy constituent such as biochanin-A was administered to a human to check LDL cholesterol level and the impact on atherosclerosis. It was shown to be efficacious to co-administer plant sterol and isoflavones ( Waggle, Potter and Henley, 2003).

**2.2.3 Obesity**

Obesity has been the most serious global health concern that is rapidly turning into an outbreak, currently affecting both developing and developed countries to varying degrees. Despite the fact that obesity and overweight are on the rise in modern society, there are no pharmacological treatments available. As a result, both researchers and health-care systems must prioritise the development of safe and effective treatments for obesity.

In HFD-induced obesity, oral treatment of biochanin-A significantly reduced the physiological changes that have occurred during trace element metabolism. This could be due to the inhibition of pathological mechanisms that derange trace elements, possibly by reverting hyperglycemia and insulin resistance and changing hepcidin and HO-1 levels. These findings strongly suggest that biochanin-A has therapeutic potential in the treatment of obesity and the prevention of cardiovascular disease ( Antony Rathinasamy *et al.*, 2020). Biochanin-A enhanced the expression of PPAR-α and its regulatory proteins in the liver by stimulating the transcriptional activation of PPAR-α in vitro. In the livers of obese mice, biochanin-A treatment increased the recovery of metabolites involved in phosphatidylcholine production, lipogenesis, and beta-oxidation. Biochanin-A also inhibited the expression of glucose 6-phosphatase and pyruvate kinase, two enzymes involved in gluconeogenesis. In diet-induced obesity, biochanin-A modulated lipid and glucose metabolism, improving metabolic abnormalities such as hepatic steatosis and insulin resistance ( Park *et al.*, 2016). Furthermore, biochanin-A administration in obese rats had a higher therapeutic effect, returning the altered parameters to near-normal levels. Biochanin-A up-regulated the Nrf-2 pathway while suppressing the NF-κB cascade, increasing the activity and mRNA expressions of enzymatic antioxidants. By activating the Nrf-2 pathway and inhibiting NF-κB activation, biochanin-A may reduce obesity and its related cardiomyopathy by decreasing oxidative stress and inflammation ( Rani A, *et al.*, 2021).

Biochanin-A promotes AMPK signalling in C3H10T1/2 MSCs, leading to upregulation of brown fat adipocyte. According to the findings, biochanin-A treatment improves mitochondrial biogenesis and lipolysis, modulating the thermogenic process. Biochanin-A improves energy expenditure by boosting mitochondrial respiration while preserving the functional mitochondria. These data imply that biochanin-A might be a new antiobesity drug ( Rahman *et al.*, 2021). Leptin is a hormone that regulates energy intake and body weight. Leptin resistance has been identified as a significant component in the development of obesity in recent studies. Endoplasmic reticulum (ER) stress, induced by the development of unfolded protein in the ER, causes leptin resistance. Biochanin-A decreased the ER stress related cell death in neuronal cells, restricted the glucose-regulated protein expression and modified the leptin signalling induced by ER stress. These findings imply that biochanin-A may have pharmacological characteristics that might reduce ER stress and hence alleviate leptin resistance ( Horiuchi *et al.*, 2021). A study deals with the evaluation of the cholesterol esterase inhibitory activity of biochanin-A using in silico docking approach. Biochanin **-**A contributed cholesterol esterase inhibitory activity, these molecular docking analyses could lead to the further development of potent cholesterol esterase inhibitors for the treatment of obesity ( Sivashanmugam *et al.*, 2013).

### 2.3 Cardiovascular disorders

Asian countries usually presented lower cardiovascular disease (CVD) mortality rates as it has very different dietary patterns from that of Western countries. Benefits over cardiovascular disease risk is an appreciated capability of soy protein and isoflavones. Isoflavones restore the disrupted endothelial function ( Sacks *et al.*, 2006; A. Gil-Izquierdo *et al.*, 2012; Sathyapalan *et al.*, 2018). By the year 1999, the US Food and Drug Administration (FDA) permitted to give soy protein-enriched foods as a protective agent in coronary heart disease routinely to lower the risk of cardiovascular disease ( U.S. FDA, 1999).

Biochanin-A mitigated myocardial injury by inhibiting the anti-inflammatory pathway, TLR4/NF-kB/NLRP3 signalling. It perfected the injury area and stopped the release of aspartate transaminase (AST), creatine kinase (CK-MB) and lactic dehydrogenase (LDH) enzyme. Biochanin-A further decreased inflammatory cytokines and protected rats from myocardial infarction ( Bai *et al.*, 2019). Reverse cholesterol transport (RCT) stimulated by biochanin-A and lowered pro-inflammatory cytokines make it a remarkable drug of choice in managing atherosclerotic cardiovascular disorder ( Yu *et al.*, 2020). Treatment with biochanin-A in isoproterenol-induced myocardial infarction rats normalized anti-oxidant levels and produced cardio-protective effects by controlling lipid peroxidation and detoxifying enzyme systems ( Govindasami *et al.*, 2020). In a study of screening various flavonoids, it was found that biochanin-A produces vasodilation in rat aorta via a mechanism of inhibiting the L-type calcium channel by interfering cGMP pathway of the coronary artery and can be of potential clinical interest in the management of the CVDs ( Migkos *et al.*, 2020).

### 2.4 Airway hyperresponsiveness

In the 19th and 20th centuries, people were drinking red clover tea or tincture (ethanolic extract) as an antispasmodic to give relief in whooping cough, measles, bronchitis, laryngitis, and tuberculosis ( Felter and Lloyd, 1999). Biochanin-A being the major constituent of red clover can act as an anti-spasmodic agent in asthma and COPD (chronic obstructive pulmonary disease) ( Ko *et al.*, 2011).

It has been proven that biochanin-A diminishes airway resistance and improves respiratory health in methacholine (MCh) induced mice. Inflammatory mediators released were under control and ovalbumin (OVA)-specific immunoglobulin E (IgE) levels were low, hence, evidencing significant effect in allergic asthma and COPD ( Ko *et al.*, 2011). Biochanin-A reduced allergic asthma in mice with histological evidence through inhibitory effects on inflammatory cytokines, cell infiltration, protein leakage into the airways and expression of haem oxygenase-1 in OVA-induced lungs (ovalbumain). The action is mediated through PPAR-γ activation ( Derangula, Panati and Narala, 2021). Biochanin-A exposed defensive effect in particulate matter with an aerodynamic diameter of 2.5 μm (PM2.5)-associated pulmonary disease rat model, it decreased cell death, the release of pro-inflammatory mediators, malondialdehyde (MDA), lactate dehydrogenase (LDH), and alkaline phosphatase (AKP) while increasing antioxidant enzymes levels ( Xue *et al.*, 2020). The risk of pulmonary and heart injury can be exacerbated by particulate matter with an aerodynamic diameter less than 10 μm (PM10). When examined in an in vitro model of lung injury caused by PM10, biochanin-A showed an anti-inflammatory effect that lessened lung injury and produced low levels of intracellular catalase and LDH. It regulates the phosphatidylinositol 3 kinase/protein kinase B (PI3K/Akt) signalling pathway and activates PI3K protein ( Li *et al.*, 2021a).

### 2.5 Osteoarthritis

Osteoarthritis is a condition that affects articular cartilage and synovial joints with structural and functional failure and diminishes the quality of life ( Hunter and Felson, 2006). It is a chronic and irreversible disease causing pain and disability. Soybean isoflavones stopped the cartilage damage in animals with an ovarian hormone deficiency, which is indicative of its effect on osteoarthritis ( Toda, Sugioka and Koike, 2020).

Biochanin-A controlled the cartilage damage by deactivating the expression of MMP, NF-κB, and activation of TIMP-1, hence effective in OA cases ( D. Q. Wu *et al.*, 2014). Biochanin-A blocked the adipocyte differentiation considerably and the level of PPAR-γ, lipoprotein lipase (LPL), and leptin and osteopontin (OPN) mRNA expression were lowered and prompted the osteoprotegerin (OPG) to put forward its ability of osteoblast differentiation stimulation and adipogenesis inhibition ( Su *et al.*, 2013). The significance of biochanin-A on the resolution of the neutrophilic inflammatory response in an antigen-induced arthritis model, using wild-type BALB/c mice showed that biochanin-A reduced the number of migrating neutrophils which was linked to decreased levels of myeloperoxidase activity, IL-1 and CXCL1, as well as the histological score in periarticular tissues. Treatment with biochanin-A improved joint dysfunction as indicated by mechanical hyper-nociception ( Felix *et al.*, 2021).

### 2.6 Inflammation

Inflammation, biological feedback of the human body to damaging stimuli, is also associated with a wide range of diseases such as “obesity, atherosclerosis, rheumatoid arthritis, and even cancer”. Isoflavones are famous for their anti-inflammatory properties. Underlining the evidence for isoflavones is required in managing chronic diseases in which inflammation plays a vital role. Isoflavones that are an assured agent in various inflammatory diseases, show exciting anti-inflammatory effects proven in animal and human studies through better anti-oxidant properties, reduced pro-inflammatory enzymes, and NF-κB regulation ( Su *et al.*, 2013).

Biochanin-A repressed LPS induced TNF-α and IL-8 production, NF-κB. Through PPAR-γ activation, biochanin-A displayed an anti-inflammatory effect hence, can be considered as an agent in the therapeutic management of inflammatory cardiovascular disease ( Su *et al.*, 2013). Biochanin-A is considered an anti-inflammatory agent with regards to its inhibitory effect in the release of nitric oxide (NO) production by LPS (Lipopolysaccharide), IkB kinase (IKK) activity, and NF-κB activation and lowered IL-6, IL-1β, and TNF-α production in RAW264.7 cells ( Kole *et al.*, 2011). It competes with the inflammation by impeding release of pro-inflammatory cytokines and modulating NF-κB and MAPKs pathways.

### 2.7 Anti-oxidant activity

Antioxidant rich foods may lower the chance of developing a number of ailments including heart disease and certain cancers. Free radicals are removed from cells by antioxidants, which minimizes oxidation related damage in the body. Biochanin **-**A being a good natural anti-oxidant can produce various health benefit in human biological system.

Water-soluble urban particulate matter is a major lung toxicant shown to induce oxidative damage in human alveolar basal-epithelial cells. Biochanin-A tested in this model produced a protective effect by increasing anti-oxidant markers such as catalase, superoxide dismutase and glutathione. The malondialdehyde (MDA) and nitric oxide (NO) levels were found to be reduced and mitigated the lung injury by regulating MEK5/extracellular signal-related kinase 5 (ERK5) nuclear factor-erythroid factor 2-related factor 2 (Nrf-2) pathway ( Xue *et al.*, 2021). Several anti-oxidant assays have shown biochanin-A to be a powerful free radical scavenger molecule with its activity comparable to ascorbic acid. Biochanin-A when assessed in anti-oxidant assays such as nitric oxide scavenging activity assay, 1,1-diphenyl-2-picryl hydrazyl (DPPH), 2,2′-azinobis-3-ethylbenzothiazoline-6-sulfonic acid (ABTS), ferric reducing antioxidant power (FRAP), hydroxy-radical activity assay, superoxide anion scavenging activity, hydrogen peroxide radical assay, metal ion chelating activity and phosphomolybdenum assay, it was observed that biochanin-A is able to produce efficient free radical scavenging ability in a dose reliant manner ( Vennila L, Asaikumar L, Sivasangari S, Jayaraj D, 2019).

### 2.8 Hepatotoxicity

The hepatoprotective abilities of biochanin-A were explored in carbon-tetrachloride hepatotoxicity animal model. The anti-oxidant and anti-inflammatory capacity of biochanin-A influence the elevated hepatic enzyme level, such as AST, ALP, ALT, bilirubin, etc., and found to be a promising molecule in hepatotoxicity models ( Breikaa *et al.*, 2013b).

### 2.9 Anti-bacterial activity

Natural isoflavones have exposed anti-microbial activity in various studies. The specific inhibitory action of biochanin-A was explored against possible *Clostridium* spp. bacterial infections of the human digestive system and was found to be beneficial to the human microbiota. “Clostridium tertium and clostridium clostridioforme” had exhibited high sensitivity to biochanin-A with a minimum inhibitory drug concentration of 0.13 mM whereas, Lactobacillus spp. or bifidobacteria showed resistant activity ( Flesar *et al.*, 2009; Sklenickova *et al.*, 2010). Out of many isoflavones, biochanin-A exhibited a growth inhibitory effect against a number of Gram-positive and Gram-negative bacteria ( Hummelova *et al.*, 2015). Augmenting the immune response of host is a novel strategy to fight against microbial infections. Biochanin-A has proven its effect as intra-cellar and extra-cellular bactericidal on HeLa cells and inhibited the Salmonella spp. infection through mTOR/AMPK/ULK1 pathway ( Zhao *et al.*, 2018a). Biochanin-A was the most effective Chlamydia spp. growth inhibitor among the various isoflavones tested, with an IC _50_ of 12 μM on Chlamydia pneumoniae inclusion counts and 6.5 μM on infectious progeny generation ( Hanski *et al.*, 2014). Biochanin-A by moderating the alterations associated with starch fermentation ex vivo, may be an efficient alternative to antibiotics for mitigating sub-acute rumen acidosis (SARA), according to a research to investigate the effect of biochanin-A on amylolytic bacteria and rumen pH during a SARA challenge ( Harlow *et al.*, 2021). The effects of biochanin-A were similar to that of monensin, an antibiotic that is used conventionally for SARA treatment in cattles. The results indicate that biochanin-A may be an effective alternate to antibiotics for alleviating SARA in cattles ( Harlow *et al.*, 2021).

### 2.10 Neurological disorders

Neurological disorders hampering the brain and nervous system are associated with a wide group of disorders as well as varying pathophysiology and symptoms. Inflammation is thought to be one important pathogenesis to cause peripheral (neuropathic pain, fibromyalgia) and central nervous systems disorders (e.g., Parkinson's disease, ischaemia, and traumatic brain injury, etc.) ( Skaper *et al.*, 2018). Multiple sclerosis (MS) is another chronic inflammatory neurodegenerative disease of the central nervous system. In a study conducted to explore the positive impacts of biochanin-A on cuprizone (CPZ)-induced MS model on mice, found that biochanin-A was able to modify the neurological harm of the condition with five weeks of treatment period. When compared to the CPZ group, biochanin-A boosted up the spatial memory in the Y-maze and recognition memory in the novel arm discrimination task (NADT) and novel object recognition task (NORT) of the animals ( Aldhahri *et al.*, 2022).

**2.10.1 Cerebral ischaemia**

Two key pathways in the development of cerebral ischaemia/reperfusion damage are oxidative stress and neuroinflammation. Biochanin-A pretreatment on experimental animals induced with stroke, showed that the neurological deficiency is improved and the size of neural infarct and brain oedema was reduced. Biochanin-A reduced oxidative stress in the brain by augmenting SOD (superoxide dismutase) and GSH-Px (glutathione peroxidase) and repressing MDA (malondialdehyde) levels. The neuroprotective effects of biochanin-A might be attributed to the activation of the Nrf2 pathway and suppression of the NF-κB pathway ( Guo *et al.*, 2019). An L-glutamate-induced cytotoxic PC12 cell line when treated with biochanin-A exposed a protective effect by reducing cytotoxicity. It aided the release of glutathione while stopping LDH, caspase-3 effects, and act as an anti-apoptotic agent to produce neuroprotective activity ( Tan *et al.*, 2013). Middle cerebral artery occlusion (MCAO) subjected animals treated with biochanin-A, aiming to produce a protective effect against cerebral ischaemia/injury, presented with suppression of inflammatory response like TNF-α and IL-1β levels, MPO activity, and downregulation of p38 signalling (W. Wang *et al.*, 2015). The elevated level of glutamate may be the principle cause that leads to cerebral ischaemia. By screening various phytomolecules, biochanin-A was found to be the most effective GOT (glutamate oxaloacetate transaminase) gene expression inducer in neural cells to alleviate ischemic injury. The glutamate induced cell death was lowered by biochanin-A administration, which was also proven when tested in GOT knock-down model as it did not have any protective effect. The ischaemic stroke presented animals were injected with biochanin-A and experienced a high level GOT protein in their brain tissues. Biochanin-A diminishes the stroke volume and amended the sensory motor abilities ( Khanna *et al.*, 2017).

**2.10.2 Parkinson’s disease**

Parkinson’s disease (PD) is related to the degeneration of dopaminergic neurons in the SNpc. Oxidative stress in connection with the neurodegenerative symptoms has been found in this condition. Studies on lipopolysaccharide (LPS)-injected animals revealed that treatment with O-methylated biochanin-A amended the behavioural patterns of animals, stopped the dopamine neuronal loss, and prevented the harmful microglia activation. Biochanin-A additionally blocked the activation of NADPH-oxidase, MDA formation, SOD, and GPx actions in the brain preferring its choice as an anti-oxidant in PD management (J. Wang *et al.*, 2015). Neurodegeneration due to inflammatory response through activating microglia is one of the reason in PD pathophysiology. It is found in rat mesencephalic neuron-glia cultures treated with biochanin-A, decreased dopamine uptake, and blocked LPS-related microglia activation. Low levels of TNF release, NO/SO release is associated with a protective effect towards LPS induced neurodegeneration ( Chen, Jin and Li, 2007). While proving the anti-inflammatory ability of biochanin-A on LPS-treated mice BV 2 microglial cells observed that the levels of TNF-α, IL-1β, nitric oxide, and ROS were lowered. Biochanin-A influences pro-inflammatory responses induced by LPS and produces a protective effect on glial cells ( Wu *et al.*, 2015). Additionally, it up-regulated PPAR-γ levels, limited NF-κB release, and acts as an anti-inflammatory agent ( Zhang and Chen, 2015). Increased neonatal iron supplementation (120 μg/g body weight) to the rats may contribute to the pathogenic mechanism of PD, and iron and rotenone co-treatment may worsen neurochemical and behavioural impairments by generating a redox imbalance. Biochanin-A had considerably lowered the malondialdehyde levels and significantly improved the glutathione levels in the substantia nigra amongst iron and rotenone co-treated male and female rats. It reduced the behavioural impairments by reducing striatal dopamine depletion. Furthermore, biochanin-A may protect dopaminergic neurons by preserving redox equilibrium ( Yu *et al.*, 2017). In another study where iron co-treated with 1-methyl-4-phenylpyridinium (MPP ^+^), it raised the level of superoxide in microglia via p38 mitogen-activated protein kinase (MAPK) stimulation and resulted in deteriorated neurochemical and behavioral features of animals. Biochanin-A has been shown to repress the activation of p38 MAPK ( Li *et al.*, 2021b).

**2.10.3 Alzheimer's disease**

The neuroprotective ability of various phytoestrogenic isoflavones including biochanin-A was screened against oxidative stress-induced cell death in the HCN 1-A (human cortical cell line) maintained in culture using the Alzheimer's disease-associated hydrogen peroxide (H _2_O _2_) model. Due to the anti-oxidant capacity of isoflavones, the concentration-reliant reduction in neuron viability by H _2_O _2_ was prevented ( Occhiuto *et al.*, 2009). The neuroprotective benefits of biochanin-A as a possible alternative to oestrogen replacement treatment, against Aβ25–35 (amyloid beta) related toxicity, as well as its putative modes of action in PC12 cells, were investigated. In the presence of biochanin-A, the effects of Aβ25–35 were considerably reversed as it lowered cell death, LDH release, and cellular caspase activity, reducing the cytotoxic impact of the Aβ25–35 protein. Furthermore, it was found that in the presence of biochanin-A, there was decreased cytochrome-c and Puma (p53 up-regulated modulator of apoptosis) expression, along with reduced Bcl-2/Bax and Bcl-xL/Bax ratio ( Tan and Kim, 2016).

The neuroprotective effects of biochanin-A on LPS-induced dopaminergic neuron injury in in vivo and in vitro models, as well as the molecular processes involved were explored. Biochanin-A therapy for 21 days substantially reduced behavioural impairment in PD rats, lowered dopaminergic neuronal loss, and suppressed microglia activation in LPS-induced PD rats. Biochanin-A protects LPS-induced PD rats, and the mechanisms are thought to be related to the suppression of the inflammatory response and the MAPK signalling pathway. Biochanin-A prevented primary microglial activation and protected dopaminergic neurons, reduced the amount of nitric oxide, IL-1, and TNF-α in supernatants, and suppressed the formation of reactive oxygen species ( Wang *et al.*, 2016). Rats lesioned with the PD-related neurotoxin 6-OHDA (6-hydroxydopamine) showed less motor impairment after receiving isoflavone-rich soy extract. These results imply that plant-derived isoflavones might be used as a dietary supplement to prevent the onset of Parkinson's disease in at-risk patients and to reduce neurodegeneration in the brains ( de Rus Jacquet *et al.*, 2021).

The behavioural and neurochemical effects of biochanin-A among cognitive-impaired animals in scopolamine-induced amnesia and naturally occurring aged animal amnesia models were assessed. In exteroceptive behavioural paradigms such as the elevated plus maze and the passive shock avoidance paradigm, biochanin-A decreased the transfer latency and increased the step through latency considerably in scopolamine-treated and natural aged mice. Acetylcholinesterase activity was found decreased in a dose-reliant manner amongst biochanin-A treated animals. A high level of GSH suppressed the pyknotic neurons formation, noradrenalin and dopamine expression thereby revealed the protective ability of biochanin-A against Alzheimer's disease ( Biradar, Joshi and Chheda, 2014). The enzyme beta-site amyloid precursor protein cleaving enzyme-1 (BACE1) is involved in the aberrant synthesis of the amyloidogenic peptide Aβ, and is one of the primary causes of Alzheimer's disease (AD). BACE1 is found to be a crucial target protein in the identification of novel strategies to minimize and prevent Alzheimer's disease. Biochanin-A non-competitively inhibited BACE1 with an IC _50_ value of 28 μM. With a binding energy of −8.4 kcal/mol, biochanin-A might strengthen the chemical's strong interaction with the allosteric site of BACE1, leading to more effective BACE1 inhibition ( Youn *et al.*, 2016).

In postmenopausal women, oestrogen insufficiency is a major risk factor for Alzheimer's disease. In the Morris water-maze test, chronic treatment with biochanin-A replicated the capacity of β-estradiol (E2) to restore learning and memory deficits in ovariectomized (OVX) rats. Biochanin-A also lowered MDA levels and increased SOD and GSH-Px enzyme levels eventually produced neuro-protective effect and can be used to treat memory loss in postmenopausal women who are suffering from an oestrogen imbalance ( Zhou *et al.*, 2021).

**2.11 Anti-fibrotic activity**

Studies have been conducted to explore the role of biochanin-A in idiopathic pulmonary fibrosis (IPF), a chronic inflammatory disease characterised by fibrotic cascade events such as epithelial-mesenchymal transition, synthesis of extracellular matrix, and collagen formation in the lungs. The study was conducted on LL29 cells (lung fibroblast from IPF patient), NHLF (normal human lung fibroblast), and DHLF (diseased human lung fibroblast). Furthermore, the research focused on evaluating the effect of biochanin-A in bleomycin-induced pulmonary fibrosis. Biochanin-A treatment produced increased levels of Smad7 expression and decreased Smad2mRNA expression in cell lines, suggesting that biochanin-A contributes to pulmonary fibrosis by inhibiting TGF-β/Smad signalling. In vivo results of the research revealed that lung index was increased in the bleomycin-induced pulmonary fibrosis group (BML) and biochanin-A reversed the same. The study proved that via modulating the TGF-β/Smad Pathway, biochanin-A prevented the onset and progression of pulmonary fibrosis ( Andugulapati *et al.*, 2020).

Biochanin-A was investigated for its antifibrotic effects on a rat liver model wherein hepatotoxicity was induced through intraperitoneal chloroform. Hepatic fibrosis is the consequence of the wound-healing action of the liver which results in the accumulation of high fibrous scar tissue. The fibrotic lesions in the biochanin-A treated group were found to be minimum, and it also decreased α-SMA levels which confirms its anti-fibrotic effect. The study observed that biochanin-A improved blood flow and have good antioxidant properties. Biochanin-A decreased the TNF-α and NO levels. Thus, biochanin-A in the liver has anti-fibrotic properties by reducing oxidative stress while preserving hepatic function ( Breikaa *et al.*, 2013a).

The several pharmacological models explained in the review are collectively summarized in Table 2 and Table 3 emphasizing the model either in vitro or in vivo along with the dose of biochanin-A used in the study and the molecular mechanism behind the activity.

**Table 2.** Pharmacological findings of biochanin-A validated via in vivo experiments. (↑increase, ↓decrease, × inhibit, + activation).

Pharmacological application	Study model	Dose and route used	Findings	Reference
**Diabetes**	STZ-rat model	10 mg/kg bodyweight per os (p/o)	HbAIc level ↓ Glucose tolerance, Insulin resistance ↓	( Harini, Ezhumalai and Pugalendi, 2012)
		10–15 mg/kg p/o	Glucose digestion ↑ HbA1C levels ↓ Serum visfatin amount ↑	( Azizi, Goodarzi and Salemi, 2014)
		10–15 mg/kg p/o	FBS ↓ hyperglycemia-induced free radicals	( Sadri *et al.*, 2017)
		15 mg/kg p/o	Nesfatin-1 ↑ Insulin ↑ FBG level ↓	( Eskandari Mehrabadi and Salemi, 2016)
		10–15 mg/kg p/o	Adiponectin ↑ insulin ↑ serum resistin ↓ serum adiponectin ↑	( Salemi *et al.*, 2018)
		0.1, 1 and 5 mg/kg intraperitonial (i/p)	Mechanical allodynia ↓ Hyperalgesia ↓	( Chundi *et al.*, 2016)
		10–15 mg/kg p/o	The concentrations of VEGF, TNF-α and IL-1β in retina ↓ blood sugar ↓ inflammation ↓ angiogenesis ↓	( Mehrabadi *et al.*, 2018)
		10–20 mg/kg/day p/o	Diabetic nephropathy × Renal TGF-β expression↑	( Ahad, 2013)
	db/db diabetic mice model	10–50 mg/kg/day red clover extract (Red clover extract containing 9.6% biochanin-A) p/o	Hepatic PPARα/γ stimulation ↑ Hepatic fatty acid synthase levels ↓	( Qiu *et al.*, 2012b)
	STZ-diabetic C57BL/6 mice	1 mg/kg/day p/o	Hepatic PPARα ↑ Lipid profile ↓ Glucose levels ↓	( Qiu *et al.*, 2012a)
	High fat diet + streptozotocin	10, 20 and 40 mg/kg	Glucose tolerance ↓ Insulin resistance ↓ Insulin sensitivity, SIRT-1 expression ↑	( Oza and Kulkarni, 2018)
	Streptozotocin- Nicotinamide rat model	5 mg/kg	Fasting blood glucose ↓ Body weight ↑	( Ghadimi *et al.*, 2019)
**Dyslipidaemia**	HFD Mice model	30mg/kg/day,60mg/kg/day, and 120mg/kg/day	LDL,total cholesterol ↓ Lipoprotein lipase and hepatic triglyceride lipase ↑	( Xue *et al.*, 2017)
**Obesity**	HFD-induced obese rats.	p/o	HFD induced trace elements metabolism ↓ Glucose, insulin, ferritin, total cholesterol, phospholipids, free fatty acids ↓	( Antony Rathinasamy *et al.*, 2020).
	C57BL/6 mice HFD	0.05% biochanin-A p/o	PPAR ↑ glucose 6-phosphatase and pyruvate kinase × Obesity-induced hepatic steatosis and insulin resistance ×	( Park *et al.*, 2016)
**Cardiovascular disorders**	Transient coronary ligation in Sprague-Dawley rats	12.5, 25 and 50 mg/kg intragastrically	TLR4/NF-kB/NLRP3 signalling × AST, CK-MB, LDH enzyme releases ↓	( Bai *et al.*, 2019)
	apoE-/- mice fed with Western diet.	50 mg/kg intragastrically	pro-inflammatory cytokines ↓	( Yu *et al.*, 2020)
	isoproterenol-induced MI rats	10 mg/kg body weight subcutaneously	antioxidant levels ↑ lipid peroxidation and detoxifying enzyme systems ↓	Govindasami *et al.*, 2020
**Airway hyper responsiveness**	Ovalbumin-Induced Airway Hyperresponsiveness BABL/c mice model	100 μmol/kg, p/o	Total inflammatory cells ↓ Eosinophils ↓ Neutrophils ↓ Th1-released IL-2 & TNF-α ↓ Th2-released IL-4 & IL-5 ↓	( Ko *et al.*, 2011)
	Male Hartley guinea pigs	10–30 μm	baseline tension and cumulative OVA-induced contractions in isolated sensitized guinea pig tracheal × degranulation of mast cells× inflammation ↓	( Ko *et al.*, 2011)
	PM2.5-induced lung toxicity in SD rats	5, 50,100 mg/kg intragastric administration	cell death ↓ release of pro-inflammatory mediators, MDA, LDH, AKP ↑ antioxidant enzymes levels ↑	( Xue *et al.*, 2020).
**Osteoarthritis**	Osteoarthritis rabbit model	5–25 mM intra-articular injection	Cartilage damage ↓ MMP, NF-κB × TIMP-1 +	( D. Q. Wu *et al.*, 2014)
**Anti-microbial activity**	BALB/c mice+ intragastric administration of *Salmonella*	2 μg/mL	Salmonella infection × AMPK/ULK1/mTOR pathway	Zhao *et al.*, 2018a)
**Neurological disorders:**				
Cerebral ischemia:	C57BL/6 male mice middle cerebral artery occlusion induced Focal cerebral ischemia	5,10 mg/kg intraperitoneal	GOT protein expression ↑ stroke lesion volume ↓	( Khanna, Stewart, Gnyawali, Harris, Balch, Spieldenner, Chandan K. Sen, *et al.*, 2017)
	Ischemic stroke Rat model	10, 20, or 40 mg/kg/day	infarct size and brain edema ↓ SOD,GSH-Px ↑ MDA,NF-κB × Nrf2 nuclear translocation, HO-1 +	Guo *et al.*, 2019)
	Middle cerebral artery occlusion (MCAO) rat model	10, 20, or 40 mg/kg/day	inflammatory response TNF-α and IL-1β levels, MPO activity↓ p38 signalling ↓	( Wang *et al.*, 2015b)
Parkinson’s disease	SD Rats with Iron and Rotenone Co-treatment	30 mg/kg	malondialdehyde ↓ glutathione levels ↑ striatal dopamine depletion↓ behavioural impairments ↓ maintains redox equilibrium	( Yu *et al.*, 2017)
	C57BL/6 with enhanced neonatal Iron and MPTP co-treatment	10–60 mg/kg	microglial p38 MAPK activation × behavioral and neurochemical deficits ↑	( Li *et al.*, 2021b)
Alzheimer's disease	LPS-induced PD rats	12.5, 25, 50 mg/kg	inflammatory response ↓ MAPK signalling pathway × nitric oxide, IL-1, and TNF-α	( Wang *et al.*, 2016).
	scopolamine-treated mice and natural aged cognitive deficit mice	40 mg/kg	AchE activity ↓ GSH ↑ thiobarbituric acid, ROS ↓ pyknotic neurons ↓	( Biradar *et al.*, 2014)
	ovariectomized (OVX) rats	5,20,60 mg/kg	restore learning and memory deficits, MDA ↓ SOD and GSH-Px ↑	( Youn *et al.*, 2016)

**Table 3.** Pharmacological findings of biochanin-A established via in vitro experiments. (↑increase, ↓decrease, × inhibit, + activation).

Pharmacological application	Study model	Dose and route used	Findings	Reference
**Diabetes**	Cervical carcinoma (HeLa) cells	EC _50_ = 1–4 mmol/L	PPAR receptor activator	( Shen *et al.*, 2006).
**Obesity**	C3H10T1/2 cells	_	Mitochondrial respiration ↑ AMPK signalling +	( Rahman *et al.*, 2021)
	Neuronal cells	_	ER stress ↓ leptin signalling ×	( Horiuchi *et al.*, 2021).
**Airway hyper responsiveness**	OVA-Induced Tracheal Contractions In Vitro.	10–30 μm	anti-spasmodic agent	( Ko *et al.*, 2011)
**Osteoarthritis**	IL-1β induced rabbit chondrocytes	5, 25, 50 μM	Cartilage damage ↓ MMP, NF-κB × TIMP-1 +	( D. Q. Wu *et al.*, 2014)
	Primary Rat Adipose-Derived Stem Cells (ADSCs).	0.3 μM	Adipocyte differentiation × PPARγ, LPL, OPN and leptin ↓ OPG ↑ osteoblast differentiation ↑ adipogenesis ×	Su *et al.*, 2013)
**Inflammation**	ADSCs	0.1–1 μM	cytoplasmic lipid droplet accumulation × PPAR-γ × Runx2, OPG, RhoA protein, OCN ↑	( Su *et al.*, 2013)
	RAW 264.7 and HT-29 cell lines	100 μM and 50 μM	NO production, LPS, IKK activity × NF-κB + IL-6, IL-1β, and TNF-α production in RAW264.7 cells ↓ cell proliferation in HT-29 cell line ×	( Kole *et al.*, 2011)
**Anti-microbial activity**	Potential bacterial pathogens of the human digestive system	0.13mM 0.26–0.51 mM	Clostridium tertium, clostridium clostridioforme ×	( Flesar *et al.*, 2009; Sklenickova *et al.*, 2010
	in vitro antibacterial activity	16 to 128 μg/mL	Growth inhibitory effect to gram-positive and gram-negative bacteria	( Hummelova *et al.*, 2015)
	*C. pneumoniae, C. trachomatis*	12 μM & 6.5 μM buccal formulation	Chlamydia growth × Antichlamydial action +	( Hanski *et al.*, 2014).
	HeLa cells/Macrophages	_	Salmonella infection × AMPK/ULK1/mTOR pathway	Zhao *et al.*, 2018a)
**Neurological disorders:**				
Cerebral ischemia:	Mouse hippocampal HT4 neural cells or primary cortical neurons	25 and 50 μM	GOT mRNA expression ↑ glutamate-induced cell death ×	( Khanna, Stewart, Gnyawali, Harris, Balch, Spieldenner, Chandan K. Sen, *et al.*, 2017)
	L-glutamate-induced cytotoxic PC12 cell line	1, 10, 50, and 100 μM	cytotoxicity ↓ glutathione ↑ LDH, caspase-3	( Tan *et al.*, 2013)
Parkinson’s disease	Rat mesencephalic neuron-glia cultures	12.5, 25, 50 mg/kg *i.p.*	NADPH-oxidase, MDA formation, SOD, and GPx × dopamine neuronal loss ↓	( Wang *et al.*, 2015a)
	BV2 microglial cells	1.25, 2.5, 5 μM	LPS related microglia activation × TNFα, IL-1β, nitric-oxide, and ROS ↓	Chen *et al.*, 2007)
	LPS treated mice BV 2 microglial cells	1.25, 2.5, 5 μM	TNFα, IL-1β, nitric oxide, and ROS ↓ MAPK signalling ×	( WU *et al.*, 2015)
	BV2 Microglia	5, 10, 20 μM	PPAR-γ levels ↑ NF-κB release ×	( Zhang and Chen 2015)
Alzheimer's disease	HCN 1-A cell line with H2O2 model	0.5, 1 and 2 μg/mL	H2O2 induced neurotoxicity ↓	( Occhiuto *et al.*, 2009)
	PC12 cells with β-Amyloid-Induced Neurotoxicity	100 μM	Amyloid beta induced cell toxicity ↓ cytochrome-c and Puma Bcl-2/Bax ↓	( Tan and Kim 2016)
	Microglial cells	1.25, 2.5, 5, 10, 15, 20, 25, 30 μM	microglial activation × nitric oxide, IL-1b, and TNF-α ↓ ROS × MAPK signaling pathway ×	( Wang *et al.*, 2016)
	In-vitro BACE-1 activity assay	28 μM	BACE1 × strong binding between the chemical and the allosteric site of BACE1	

## 3. Clinical trial on biochanin-A

A clinical trial is underway to check the effect of red clover extract including biochanin-A among post-menopausal women with osteopenia. The results are yet to be concluded ( University of Aarhus, 2015). The clinical trial data are summarized in Table 4

**Table 4.** Clinical trial on biochanin-A.

Sl no.	Study design	Title of the study	Objective	Comparison to:
1	Double-blind parallel, randomized intervention trial ( University of Aarhus, 2015)	Postmenopausal women with osteopenia (low bone mineral density)	Effects of daily intake of fermented red clover extract on oestrogen dependent bone mineral resorption	Placebo

## 4. Limitation of biochanin-A

Biochanin-A is a poorly soluble bioflavonoid, which prevents its oral absorption, despite having a rapid clearance and a broad apparent volume of distribution. The bioavailability of biochanin-A is poor. It was reported that biochanin-A undergoes extensive metabolism. The pharmacological value of biochanin-A was limited by its poor water solubility and low bioavailability. Numerous attempts have been made to increase the solubility and bioavailability of biochanin-A, including the use of dispersants, silver nanoparticles, liposomes, different film formulations for buccal delivery, nanostructured lipid carriers with and without polyethylene glycol, and cyclodextrin inclusion complexes. Esters of biochanin-A and carbamate ester derivatives have also been developed, and they have higher metabolic stability than biochanin-A. Thus, several attempts are being made worldwide to enhance biochanin-A’s solubility and bioavailability without compromising its effectiveness ( Yu *et al.*, 2019).

## 5. Conclusion

This review discusses various pharmacological applications of biochanin-A focussing on the molecular pathway that might be responsible for its beneficial action. Biochanin-A might be able to modify various systems of the human body like the cardiovascular system, CNS, respiratory system, etc. It has a remarkable effect on hormonal cancers and other types of cancers. The growing amount of research on biochanin-A in breast, lung, colon, prostate, pancreatic cancers is an illustration of its impact in medicine. Through modulating oxidative stress, SIRT-1 expression, PPAR-γ receptors, and other multiple mechanisms, biochanin-A produces anti-diabetic action. The diverse molecular mechanistic pathways involved in the pharmacological ability of biochanin-A indicate that it is a very promising molecule and have the potential to stimulate drug development for multiple disorders.

## Data Availability

No data are associated with this article.
