# Supplementary material for: Mechanisms Behind the Pharmacological Application of Biochanin-A: A review
Source: F1000Res. 2023 Dec 11;12:107. Originally published 2023 Jan 27. [Version 3] doi: 10.12688/f1000research.126059.3 (PMC10725524; doi:10.12688/f1000research.126059.3)
Supplement: Peer review history

# Reviewer report: Reviewer response for version 3

**DOI:** 10.5256/f1000research.159627.r229121

**Published:** 2023-12-16

**Authors:** Reyaz Hassan Mir

**Affiliations:** 1 Department of Pharmaceutical Sciences, University of Kashmir, Srinagar, India

APPROVED 

 All the comments are addressed in the revised manuscript. So go ahead for the final print from my side

Is the review written in accessible language?

Yes

Are all factual statements correct and adequately supported by citations?

Yes

Are the conclusions drawn appropriate in the context of the current research literature?

Partly

Is the topic of the review discussed comprehensively in the context of the current literature?

Yes

Reviewer Expertise:

Pharmaceutical Chemistry (Natural products in drug Discovery)

I confirm that I have read this submission and believe that I have an appropriate level of expertise to confirm that it is of an acceptable scientific standard.

# Reviewer report: Reviewer response for version 2

**DOI:** 10.5256/f1000research.154748.r221215

**Published:** 2023-11-22

**Authors:** Reyaz Hassan Mir

**Affiliations:** 1 Department of Pharmaceutical Sciences, University of Kashmir, Srinagar, India

The paper entitled “Mechanisms behind the pharmacological application of biochanin-A: a review by Anuranjana PV *et al*. gives an interesting perspective on the field. However, there are some minor comments which should be addressed before it is suitable for publication.

1. The title of the Manuscript needs to be updated, all the first letters should have been kept in Upper case letters as Mechanisms Behind the Pharmacological Application of Biochanin-A: A review
2. The abstract need to be reframed, you must start with your abstract by giving a brief introduction of natural products and its current role in drug discovery.
3. The first two lines of the abstract should be kept at the end of abstract
4. The Introduction part needs to be begin with natural products and the current drugs from natural products in the market, and then a brief introduction to isoflavones
5. The term potency at line No. 3 in the introduction section is specific for drugs only and biochanin-A is not a drug, so replace this term.
6. The term efficacy in the section 2.1 does not fit with this compound, so replace it with some relevant term and other places as well. This term is specific for the marketed drugs only
7. Biochanin-A is not written properly at all places, Check the correct way of writing. i.e with or without hyphen
8.  In section 2.9 i.e Antimicrobial activity, you have  mentioned only antibacterial activity only, if it is so then change the title as antibacterial activity.
9.  There is no need to write in the conclusion (Last line) biochanin-A can play a major impact in modifying several physiological functions. Remove this sentence.
10. I recommend the authors to check and update bibliography and include the most recently published papers in the field.

Is the review written in accessible language?

Yes

Are all factual statements correct and adequately supported by citations?

Yes

Are the conclusions drawn appropriate in the context of the current research literature?

Partly

Is the topic of the review discussed comprehensively in the context of the current literature?

Yes

Reviewer Expertise:

Pharmaceutical Chemistry (Natural products in drug Discovery)

I confirm that I have read this submission and believe that I have an appropriate level of expertise to confirm that it is of an acceptable scientific standard, however I have significant reservations, as outlined above.

# Response

**Published:** 2023-11-30

**Authors:** Krishnadas Nandakumar

**Affiliations:** Manipal College of Pharmaceutical Sciences, Manipal Academy of Higher Education, India

**Response to Comment 1:**

 Thank you for suggesting. The manuscript title is now modified as per the reviewer’s suggestion as “Mechanisms Behind the Pharmacological Application of Biochanin-A: A review”

**Response to Comment 2:**

 As per the reviewer’s suggestion, the authors have re-written the abstract as below;

 Natural products continue to be an important resource in novel drug discovery and serve as an inspiration for the development of many synthetic drugs. Flavonoids hold particular interest to researchers due to their remarkable structural diversity and complex biological functions. Biochanin-A is one of the prominent isoflavones found in soy (glycine max), red clover (Trifolium pratense), and alfalfa sprouts, etc., with proven potency in modulating vital cellular mechanisms in various diseases. It has been popular for ages among menopausal women in controlling symptoms. In view of the multi-targeted functions of biochanin-A, it is essential to summarize its mechanism of action in various disorders. It has shown a remarkable effect on hormone-dependent cancers like breast, prostate, and other malignancies like pancreatic, colon, lung, osteosarcoma, and glioma that have limited treatment options, biochanin-A revealed agreeable results in arresting cancer development. Biochanin-A has also shown therapeutic benefits when administered for neurological disorders, diabetes, hyperlipidaemia, and other chronic diseases/disorders.**** Through modulating oxidative stress, SIRT-1 expression, PPAR gamma receptors, and other multiple mechanisms biochanin-A produces anti-diabetic action. The safety and efficacy of biochanin-A need to be established in clinical trials involving human subjects.**** The diverse molecular mechanistic pathways involved in the pharmacological ability of biochanin-A indicate that it is a very promising molecule and can play a major impact in modifying several physiological functions. Many studies have been reported claiming its application in cancers, metabolic disorders, airway hyperresponsiveness, cardiac disorders, neurological disorders, etc. With this review, we aim to summarize the cellular and molecular mechanisms behind the various pharmacological actions of biochanin-A.

**Response to Comment 3:**

 The revised abstract removed repetitive content and modified the final sentence of the abstract following the reviewer's recommendations.

**Response to Comment 4:**

 The authors have included a brief introduction about natural products and isoflavones as suggested;

 Introduction:

 Traditionally, going back to nature has always been an answer, and in reality, natural products have been essential in the development of new drugs, particularly for infectious and cancerous disorders (Atanasov *et al.*, 2021). Natural products have been a rich source of new drugs for centuries. ‘Anticancer drugs such as Taxol (Taxus brevifolia) and Vinblastine (Catharanthus roseus) and antimalarial drugs such as quinine (Cinchona spp.) and Artemisinin (Artemisia annua)’ were all discovered from natural products and have been effective in treating these diseases (Singh *et al.*, 2021).

 Phytoestrogens are naturally occurring nonsteroidal phenolic plant compounds that can be categorized into flavonoids and non-flavonoids (Boyle *et al.*, 2003). Flavonoids encompass isoflavones, coumestans, and prenylflavonoids, while non-flavonoids comprise lignans. The major isoflavones are genistein (7,4′-dihydroxy-6-methoxyisoflavone), daidzein (7,4′-dihydroxyisoflavone), glycitein (7,4′-dihydroxy-6-methoxyisoflavone), biochanin-A (5,7-dihydroxy-4′-methoxyisoflavone), and formononetin (7-hydroxy-4´-methoxyisoflavone). Isoflavones, renowned for their estrogenic properties, are predominantly found in legumes from the Fabaceae family (Dixon and Sumner, 2003), with soybean (Glycine max) as a source of daidzein, genistein, glycitein, and red clover (Trifolium pratense) as a source of formononetin and biochanin-A.

 Isoflavones, predominantly found in soybean and soybean products serve as major dietary sources for humans. Isoflavones are recognized for their chemoprotective properties, offering an alternative therapeutic approach for various hormonal disorders, including breast cancer, prostate cancer, cardiovascular diseases, osteoporosis, and menopausal symptoms (Křížová *et al.*, 2019).

**Response to Comment 5:**

 The authors would like to change the word potency in the sentence. The sentence is written as follows;

 ‘It can benefit humans in various systems.’

**Response to Comment 6:**

 Considering the suggestion given by the reviewer, the authors would like to change the term ‘efficacy’ used in various sentences as follows;

 Section 2.1

 ‘Biochanin-A has shown efficacy against different types of cancers.’ changed to ‘Biochanin-A has prevented/ inhibited the development of different types of cancers’

 Figure 1.

 ‘The schematic diagram represents pharmacological efficacy of biochanin-A in different types of cancer.’ Changed to ‘The schematic diagram represents pharmacological actions of biochanin-A in different types of cancer’

 ‘Biochanin-A has shown efficacy against different types of cancers.’ changed to  ‘Biochanin-A has prevented the developnent different types of cancers’

 2.1.8 Leukaemia & other cancers:

 ‘This study suggests that Biochanin-A could be a novel treatment option for multiple myeloma with superior efficacy and reduced toxicity’ changed to ‘This study suggests that Biochanin-A may offer a new, more effective, and less toxic type of treatment for multiple myeloma.’

  2.10.3 Alzheimer's disease

 ‘The neuroprotective efficacy of various phytoestrogenic isoflavones including biochanin-A was screened against oxidative stress-induced cell death in the HCN 1-A (human cortical cell line) maintained in culture using the Alzheimer's disease-associated hydrogen peroxide (H2O2) model’ changed to ‘The neuroprotective ability of various phytoestrogenic isoflavones including biochanin-A was screened against oxidative stress-induced cell death in the HCN 1-A (human cortical cell line) maintained in culture using the Alzheimer's disease-associated hydrogen peroxide (H2O2) model’

 ‘Due to the anti-oxidant efficacy of isoflavones, the concentration-reliant reduction in neuron viability by H2O2 was prevented (Occhiuto et al., 2009).’ Changed to ‘Due to the anti-oxidant capacity of isoflavones, the concentration-reliant reduction in neuron viability by H2O2 was prevented (Occhiuto et al., 2009).’

**Response to Comment 7:**

 The authors would like to use a hyphen along with the term biochanin-A with A as a capital letter. We have changed the misspelled terms in the review to biochanin-A

**Response to Comment 8:**

 The authors would like to change the title to Anti-bacterial Activity as per the reviewer's suggestion

**Response to Comment 9:**

 The authors have removed the sentence as the reviewer's recommendation. The last line is modified as ‘The diverse molecular mechanistic pathways involved in the pharmacological ability of biochanin-A indicate that it is a very promising molecule and has the potential to stimulate drug development for multiple disorders.’

**Response to Comment 10:**

 The following references have been added

 Figure 1 (strucrure of biochanin-A)

 (Feng and Lai, 2023)

 Feng, Z.-J. and Lai, W.-F. (2023) ‘Chemical and Biological Properties of Biochanin A and Its Pharmaceutical Applications’, *Pharmaceutics*, 15(4), p. 1105. doi: 10.3390/pharmaceutics15041105.

 2.3 Cardiovascular disorders

 In a study of screening various flavonoids, it was found that biochanin-A produces vasodilation in rat aorta via a mechanism of inhibiting the L-type calcium channel by interfering cGMP pathway of the coronary artery and can be of potential clinical interest in the management of the CVDs (Migkos *et al.*, 2020).

 (Migkos, T. et al. (2020) ‘Biochanin A, the Most Potent of 16 Isoflavones, Induces Relaxation of the Coronary Artery Through the Calcium Channel and cGMP-dependent Pathway’, Planta Medica, 86(10), pp. 708–716. doi: 10.1055/a-1158-9422.)

# Reviewer report: Reviewer response for version 2

**DOI:** 10.5256/f1000research.154748.r197869

**Published:** 2023-08-25

**Authors:** Sai Balaji Andugulapati

**Affiliations:** 1 Division of Applied Biology, CSIR-Indian Institute of Chemical Technology, Hyderabad, Telangana, India

Need to keep ± SEM/SD

Is the review written in accessible language?

Yes

Are all factual statements correct and adequately supported by citations?

Partly

Are the conclusions drawn appropriate in the context of the current research literature?

Yes

Is the topic of the review discussed comprehensively in the context of the current literature?

Yes

Reviewer Expertise:

Cancer Biology, Pulmonary fibrosis (IPF), NASH and hepatic fibrosis

I confirm that I have read this submission and believe that I have an appropriate level of expertise to confirm that it is of an acceptable scientific standard.

# Reviewer report: Reviewer response for version 1

**DOI:** 10.5256/f1000research.138434.r161697

**Published:** 2023-07-14

**Authors:** Sai Balaji Andugulapati

**Affiliations:** 1 Division of Applied Biology, CSIR-Indian Institute of Chemical Technology, Hyderabad, Telangana, India

I have gone through the article entitled 'Mechanisms behind the pharmacological application of biochanin-A'. It was written well however, the authors need to address the following comments and need additions in a few places.

1. On page no. 3, biochanin shows therapeutic actions in various cancers, need to mention about multiple myeloma as well, because biochanin-A showed anti-MM activity, and need to describe in the blood cancer section or wherever it is appropriate. 
2. On page no. 3, the authors wrote: " *During breast cancer development the normal signalling pathways are interrupted, stimulating the refractory growth, no cell death, and progressive invasion to neighbouring tissues. Soy-rich diets in controlling hormone-dependent cancers have gained wide attention lately.*" This can happen in the majority of solid cancers, is there any specific reason to specify only breast cancer?
3. On page no. 5, the authors wrote: " *Biochanin-A has shown synergism with 5-fluorouracil (5FU) in oestrogen receptor (ER) positive breast cancer cell lines such as triple-negative breast cancer cells, MDA-MB231 and MCF7.*" But MCF7 cells are not triple-negative cells, authors need to check and re-write these sentences. 
4. On page no. 6, the authors mentioned: " *The patients with clinically significant prostate cancer were treated with red clover isoflavones such as biochanin-A before surgical intervention. Markedly, higher apoptosis was found in the treatment group, indicates cessation of prostate cancer progression in low- moderate grade malignancy (Jarred et al., 2002).*" I failed to understand if is a clinical study or in vitro study, the first sentence shows as a clinical study, but the second sentence is depicted as an in vitro study. Please clarify, such confusion is there in multiple places...please correct it.
5. On page no. 6, the authors wrote: " *Soy-rich foods may reduce the likelihood of lung malignant growth in the general population, according to epidemiology researches...*" Maybe it should be written as: "Soy-rich foods may reduce the occurrence of getting cancer in the general population, according to epidemiology researches", please check it.
6. On page no. 9, it's written that *"biochanin-A exhibits neuroprotective effect*" and gave references for cancer studies, please give references for neuroprotective not for neuro cancer. 
7. Few of the references are not suitable, please check it.
8. Biochanin-A is known to show anti-fibrotic activity as well, especially lung (IPF) and liver, authors should explore that as well.
9. Adding limitations of biochanin-A would improve the manuscript.

Overall the review is good.

Is the review written in accessible language?

Yes

Are all factual statements correct and adequately supported by citations?

Partly

Are the conclusions drawn appropriate in the context of the current research literature?

Yes

Is the topic of the review discussed comprehensively in the context of the current literature?

Yes

Reviewer Expertise:

Cancer Biology, Pulmonary fibrosis (IPF), NASH and hepatic fibrosis

I confirm that I have read this submission and believe that I have an appropriate level of expertise to confirm that it is of an acceptable scientific standard, however I have significant reservations, as outlined above.

# Response

**Published:** 2023-07-29

**Authors:** Krishnadas Nandakumar

**Affiliations:** Manipal College of Pharmaceutical Sciences, Manipal Academy of Higher Education, India

Response for Comment-1:

 As per the reviewer's suggestion, the following part has been added to the content of the paper

**2.1.8 Leukemia & other cancers**

  A study has found that Biochanin-A can be used to treat multiple myeloma, which is a type of cancer that affects plasma B cells in the bone marrow. The application of Biochanin-A has been shown to reduce the level of CD38, which is one of the key therapeutic targets for combating Multiple Myeloma. It has also been found to trigger apoptosis in Multiple Myeloma cells and reduce cytokine expression. The study further explored NOD/SCID mice with U266-induced tumors. Biochanin-A treatment was found to significantly reduce tumor growth. Mechanistic studies have shown that Biochanin-A’s anti-cancer effects are achieved by modulating the NF-κB and MAPK signaling pathways. This study suggests that Biochanin-A could be a novel treatment option for multiple myeloma with superior efficacy and reduced toxicity (Jaina et al., 2022).

Response for Comment-2:

 The reason to specify breast cancer in the sentence was to give a general description of the topic under the subheading (2.1.1. Breast cancer) and elaborate on the pharmacological effects of Biochanin-A on breast cancer.

 As per the reviewer's suggestion, the text is changed as follows: “In most types of solid cancers like breast cancer, the normal signalling pathways are interrupted, stimulating refractory growth, no cell death, and progressive invasion of neighbouring tissues. Soy-rich diets in controlling hormone-dependent cancers have gained wide attention lately.”

Response for Comment-3:

 The sentence has been re-written as follows:

 Biochanin-A has shown synergism with 5-fluorouracil (5FU) in oestrogen receptor (ER) positive breast cancer cell lines such as MCF7, and triple-negative breast cancer cells such as MDA-MB231.

Response for Comment-4:

 The study was a nonrandomized, nonblinded clinical study performed in men with clinically significant prostate cancer before radical prostatectomy. The apoptosis markers in prostate tumor cells from radical prostatectomy specimens were compared among the red clover-derived isoflavones including Biochanin-A treated groups. The study is not an in vitro study. The authors would like to modify the sentence as:

 The patients with clinically significant prostate cancer were treated with red clover isoflavones such as biochanin-A before surgical intervention. The apoptosis markers in prostate tumor cells from radical prostatectomy specimens were analysed. Markedly, higher apoptosis was found in the treatment group with a dietary supplement of isoflavones, indicating cessation of prostate cancer progression in low to moderate-grade malignancy (Jarred et al., 2002).

Response for Comment-5:

 The sentence has been rewritten as suggested.

 Soy-rich foods may reduce the occurrence of cancer development in the general population, according to epidemiology research”.

Response for Comment-6:

 The sentence has been rewritten as suggested.

 Soy-rich foods may reduce the occurrence of cancer development in the general population, according to epidemiology research”.

Response for Comment-7:

 The current manuscript is revised and has suitable references quoted at appropriate places. If the reviewer feels still there is scope for improvement in this area, I request the reviewer to specify it so that it can be suitably altered.

Response for Comment-8:

 The authors would like to add a section with subheading 2.11 Anti-fibrotic activity

**2.11 Anti-fibrotic activity**

 Studies have been conducted to explore the role of biochanin-A in idiopathic pulmonary fibrosis (IPF), a chronic inflammatory disease characterised by fibrotic cascade events such as epithelial-mesenchymal transition, synthesis of extracellular matrix, and collagen formation in the lungs. The study was conducted on LL29 cells (lung fibroblast from IPF patient), NHLF (normal human lung fibroblast), and DHLF (diseased human lung fibroblast). Furthermore, the research focused on evaluating the effect of biochanin-A in bleomycin-induced pulmonary fibrosis. Biochanin-A treatment produced increased levels of Smad7 expression and decreased Smad2mRNA expression in cell lines, suggesting that biochanin-A contributes to pulmonary fibrosis by inhibiting TGF-/Smad signalling. *Invivo *results of the research revealed that lung index was increased in the bleomycin-induced pulmonary fibrosis group (BML) and biochanin-A reversed the same. The study proved that via modulating the TGF-β/Smad Pathway, biochanin-A prevented the onset and progression of pulmonary fibrosis (Andugulapati *et al.*, 2020).

 Biochanin-A was investigated for its antifibrotic effects on a rat liver model wherein hepatotoxicity was induced through intraperitoneal chloroform. Hepatic fibrosis is the consequence of the wound-healing action of the liver which results in the accumulation of high fibrous scar tissue. The fibrotic lesions in the biochanin-A treated group were found to be minimum, and it also decreased α-SMA levels which confirms its anti-fibrotic effect. The study observed that biochanin-A improved blood flow and have good antioxidant properties. Biochanin-A decreased the TNF-α and NO levels. Thus, biochanin-A in the liver has anti-fibrotic properties by reducing oxidative stress while preserving hepatic function (Breikaa *et al.*, 2013).

Response for Comment-9:

 The authors would like to add a separate subheading after the clinical trial section as below:

 The authors would also like to correct the number of subsections of the conclusion as 5

**4. Limitation of Biochanin-A**

 Biochanin-A is a poorly soluble bioflavonoid, which prevents its oral absorption, despite having a rapid clearance and a broad apparent volume of distribution. The bioavailability of biochanin-A is poor. It was reported that biochanin-A undergoes extensive metabolism. The pharmacological value of biochanin-A was limited by its poor water solubility and low bioavailability. Numerous attempts have been made to increase the solubility and bioavailability of biochanin-A, including the use of dispersants, silver nanoparticles, liposomes, different film formulations for buccal delivery, nanostructured lipid carriers with and without polyethylene glycol, and cyclodextrin inclusion complexes. Esters of biochanin-A and carbamate ester derivatives have also been developed, and they have higher metabolic stability than biochanin-A. Thus, several attempts are being made worldwide to enhance biochanin-A’s solubility and bioavailability without compromising its effectiveness (Yu *et al.*, 2019).
